# *In vitro* and *in vivo* antifungal effects of fluconazole in combination with *Cinnamomum verum* essential oil against *Candida* spp

**DOI:** 10.3389/fphar.2026.1864146

**Published:** 2026-07-01

**Authors:** Marina Ionela Nedea, Serena-Ștefania Velicu, Ioana Cristina Marinaş, Mihaela Buleandră, Andreea Letiția Arsene, Bruno Ștefan Velescu, Mariana Carmen Chifiriuc, Irina Gheorghe-Barbu

**Affiliations:** 1 Faculty of Pharmacy, “Carol Davila” University of Medicine and Pharmacy, Bucharest, Romania; 2 Faculty of Biology, University of Bucharest, Bucharest, Romania; 3 The Research Institute of the University of Bucharest (ICUB), Bucharest, Romania; 4 Faculty of Chemistry, University of Bucharest, Bucharest, Romania; 5 The Romanian Academy, Bucharest, Romania

**Keywords:** antifungal, anti-virulence, combination therapy, *in vivo* model, multidrug-resistant *Candida* strains, synergistic effects

## Abstract

**Introduction:**

Antifungal resistance in *Candida* species is a growing clinical problem globally, especially in immunocompromised patients. One of the alternative approaches to conventional therapies are currently based on essential oils (EOs) alone or in combination with antifungals. We aimed to evaluate the antifungal activity of *Cinnamomum verum* EO, alone and in combination with fluconazole, against reference and multidrug-resistant (MDR) *Candida* strains by *in vitro* and *in vivo* assays.

**Methods:**

The chemical composition of the EO was analyzed by gas chromatography-mass spectrometry. Antifungal activity was assessed by broth microdilution (minimum inhibitory concentration determination), while anti-adherence effects were evaluated using the microtitration with crystal violet. The fractional inhibitory concentration index and response surface approach were used to study synergistic interactions. The *in vivo* efficacy was assessed by tracking virulence factors, fungal load, and larval survival in *Galleria mellonella* model.

**Results:**

The most significant phenylpropanoid components of the EO were (E)-cinnamaldehyde and eugenol. It demonstrated high antifungal activity and significantly decreased adherence to the inert substratum. The tested EO exhibited pharmacological synergy in combination with fluconazole, especially against fluconazole-resistant *Candida auris* strains. The optimized fluconazole-EO combination decreased fungal virulence and load, as well as *G. mellonella* larval mortality.

**Conclusion:**

*C. verum* EO exhibits strong anti-virulence and antifungal effects and increases fluconazole activity, indicating its potential as an adjuvant treatment against resistant *Candida* infections.

## Graphical Abstract

**Figure:**
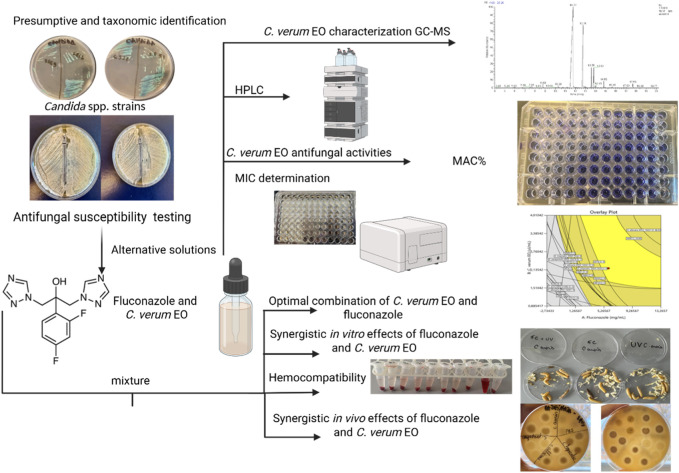

## 1 Introduction

Species belonging to the *Candida* genus are opportunistic pathogens, especially among immunocompromised patients, due to antifungal resistance. In 2022, the World Health Organization (WHO) classified several *Candida* species [*Candida albicans, Candidozyma auris* (syn. *Candida auris*), *Nakaseomyces glabratus* (syn. *Candida glabrata*), *Candida parapsilosis*, and *Candida tropicalis*] as critical or high-priority pathogens because of their widespread occurrence and ineffectiveness to currently available antifungal treatments (World Health Organization (WHO), 2022). Currently, azoles, polyenes, echinocandins, and pyrimidine analogues are the four antifungal classes used to treat candidiasis. However, the increasing number of resistant strains significantly reduces the efficacy of treatment, making clinical management more difficult and raising worries about their effects on public health (Arastehfar et al., 2020). In reaction to these difficulties interest in alternative therapeutic approaches has increased. These strategies include novel antifungal compounds, the use of combination treatments, and anti-virulence modulation strategies (anti-adherence and biofilm formation, enzyme secretion) (Ahaik et al., 2026).

Natural products, particularly those derived from aromatic and medicinal plants, have drawn more attention as potential sources of new antimicrobial chemicals. Among these, essential oils (EOs) have shown significant antifungal activity against a variety of infections, including candidiasis (Tabassum and Vidyasagar, 2013). Because of its potent biological activity, cinnamon EO is of special interest among them. Their activity is associated with major phenolic components, like cinnamaldehyde, which is listed in the European Commission’s 2008 list of aromatic compounds deemed safe for human health. These compounds exhibit antifungal properties primarily by compromising the integrity of the fungal cell membrane (inhibiting ergosterol synthesis), increasing permeability and decreasing ATPase activity (Yu, 2025; OuYang et al., 2019), causing membrane peroxidation, and ultimately inducing apoptosis by causing an accumulation of reactive oxygen species (ROS) in cells (Hu et al., 2011).

The ethnomedicine has long exploited the carminative, antimicrobial, anti-inflammatory and tonic properties of cinnamon bark and its EO (Rao and Gan, 2014; Gruenwald et al., 2010), used to treat respiratory tract infections, inflammation, gastrointestinal and metabolic illnesses. Due to its high content of bioactive phenylpropanoids, especially eugenol and cinnamaldehyde, known for their antimicrobial properties, the EO has been also used as a food preservative (Shan et al., 2007). Despite an expanding body of evidence, less is known about the anti-virulence effects of *C. verum* EO (e.g., the expression of proteolytic, lipolytic, and hemolytic enzymes essential to fungal pathogenicity) (Silva et al., 2014) and its effects on clinical, multidrug-resistant (MDR) *Candida* strains.

A viable *in vivo* assay to evaluate the fungal pathogenicity and the efficacy of different antifungal therapies is the *Galleria mellonella* infection model (Barretto et al., 2020). The functional similarities between insect and mammalian innate immune responses, low complexity of handling, high degree of reproducibility, and less ethical issues are some of this paradigm’s advantages over vertebrate models (Holt et al., 2026).

Therefore, using a combination of *in vitro* assays and the *G. mellonella in vivo* model, this study aimed to investigate the antifungal efficacy of *C. verum* EO, alone or in combination with fluconazole, against both reference strains and clinical MDR *Candida* isolates, while also examining possible synergistic interactions.

## 2 Materials and methods

### 2.1 Materials

All chemicals and reagents used in this study were analytical grade. Chemical substances included fluconazole (ROTICHROM®, Merck, Germany), dimethyl sulfoxide (DMSO), hexane, crystal violet, Triton X-100, and phosphate-buffered saline (PBS), all purchased from Sigma-Aldrich (St. Louis, MO, USA). Microbiological media included RPMI 1640 medium (Sigma-Aldrich, USA), Sabouraud Dextrose Agar (SDA, Alliance Bio Expertise, France), and CHROMagar™ *Candida* Plus (CHROMagar, France). Solvents used for chromatographic analysis included methanol and acetonitrile (HPLC grade, Merck, Germany). The *C. verum* EO, extracted from the bark of the plant, was purchased from an authorized local producer (S.C. Santo Raphael S.R.L., Bucharest, Romania), in its original packaging, accompanied by a certificate of conformity and stored in sealed glass containers at 4 °C until use. *G. mellonella* larvae (200–300 mg) were obtained from a commercial supplier and maintained under controlled conditions (30 °C, 70% relative humidity) prior to experimentation, using last instar larvae.

### 2.2 *C. verum* EO characterization

Analysis of C. verum EO was performed using a Thermo Electron system (Thermo Fisher Scientific, Waltham, MA, USA) consisting of a Focus gas chromatograph (GC) with a DB-5MS capillary column (25 m × 0.25 mm, 0.25 µm film thickness), a TriPlus autosampler, and a Polaris Q ion trap mass detector. Helium was used as the carrier gas at a flow rate of 1 mL/min. The injector and detector temperatures were set at 250 °C. The oven temperature was programmed to increase from 60 °C for three minutes to 200 °C at a rate of 10 °C/min, and then from 200 °C to 240 °C at a rate of 12 °C/min. Mass spectra were recorded at 70 eV (EI) and scanned in the 35–450 m/z range. The samples were diluted with hexane (1/100) and introduced into the injector in splitless mode. The volatile components were identified using Xcalibur® software and the NIST 11 Mass Spectral Library. An alkane standard solution for GC (C8–C20 in hexane, Sigma-Aldrich, USA) was used to determine the retention indexes (RIs). The relative percentage content of each component was calculated based on the GC peak areas, with no correction factors applied.

Additionally, the physical characterization included the measurement of the refractive index, following the methodology described by Falleh et al. (2021). The analysis was performed using a Kruss DR 201-95 digital handheld refractometer. Briefly, a drop of the *C. verum* EO was placed onto the horizontal prism, which was then secured to ensure full contact. All readings were conducted in accordance with established food-grade characterization standards to ensure accuracy and reproducibility.

### 2.3 Evaluation of fluconazole chemical stability in the presence of *C. veru*m EO by high-performance liquid chromatography (HPLC)

To evaluate the stability of the fluconazole - *C. verum* EO mixture, HPLC analysis was performed. The samples were monitored for a period of 30 days and stored under various environmental conditions: light and ambient temperature, darkness and ambient temperature, a refrigerator (4 °C) and a thermostat at 37 °C. Freshly prepared samples (time T0) were also analyzed, as well as separate controls of fluconazole ROTICHROM® (Merck, Germany) and *C. verum* EO. The fluconazole calibration curve included values between 46.65 and 746.4 μg/mL (R2 = 0.9997). Each sample was solubilized in methanol, followed by ultrasound for 10 min. Chromatographic analyses were carried out using an Agilent 1100 series HPLC system equipped with a Zorbax C18 column (100 mm × 3 mm, 3.5 µm). Detection was performed at a wavelength of 260 nm at a temperature of 22 °C. The mobile phase was an isocratic mixture of acetonitrile (20%) and a 0.1% phosphoric acid solution (80%) at a flow rate of 1 mL/min. All injected samples maintained under various conditions had a final concentration of 0.32 µg fluconazole and 0.06 µL of EO. The injection volume was 10 μL, and the retention time for fluconazole was 1.47 min (Nedea et al., 2025a).

### 2.4 *Candida* spp. strains characterization - identification and antifungal susceptibility testing by E-test method

Clinical isolates of *Candida* spp (n = 8) were obtained from different types of specimens collected from patients admitted to the National Institute of Pneumophthisiology “Marius Nasta”, Bucharest, Romania, between January and May 2024 from different isolation sources as previously described (37.5% from urine, 25% from bronchial aspirate, and respectively 12% from eschar, vaginal discharge and perianal swab) (Nedea et al., 2025b). Primary isolation was performed on SDA. Specific identification of the recovered isolates was carried out using the CHROMagar™ *Candida* Plus medium (Chromagar, Saint-Denis, France) and the automated VITEK® 2 COMPACT identification system. For comparative purposes, four well-characterized reference strains were also included in the study: *Candida albicans* ATCC 10231, *Candida auris* DSM 21092, *Candida parapsilosis* ATCC 22019 and *Candida tropicalis* DSM 7524. All strains were preserved in glycerol stocks at −20 °C until experimental procedures were conducted. The use of nosocomial isolates was authorized by the Human Research Ethics Commission of the National Institute of Pneumophthisiology “Marius Nasta” (Approval No. 12255/17 June 2024). All procedures complied with the ethical standards outlined in the Declaration of Helsinki. Antifungal susceptibility of the tested *C. auris* strains was evaluated using the E-test method to determine minimum inhibitory concentration *(*MIC) values for fluconazole, micafungin, amphotericin B, caspofungin, and flucytosine, with results interpreted according to the Clinical and Laboratory Standards Institute (CLSI) M60 guidelines as previously described (Gary, 2022; Nedea et al., 2025b).

### 2.5 Antifungal activity of *C. verum* EO against reference and nosocomial *Candida* spp. strains by MIC determination

The antifungal susceptibility testing of *C. verum* EO against reference and clinical *Candida* spp. isolates was evaluated using the broth microdilution method in RPMI 1640 medium (Sigma-Aldrich, St. Louis, MO, USA), in accordance with the standardized protocols recommended by the Clinical and Laboratory Standards Institute (CLSI, 2022) and the European Committee on Antimicrobial Susceptibility Testing (European Committee on Antimicrobial Susceptibility Testing, 2024). The EO was initially dissolved in DMSO to obtain a stock solution of 20 μL/mL. Subsequently, two-fold serial dilutions under a concentration range of 10 - 0.009 μL/mL in RPMI 1640 medium were tested. After incubation, fungal growth was evaluated by measuring absorbance at 620 nm with a microplate reader (Thermo Fisher Scientific, Waltham, MA, USA) and the determination of the MIC values after the blank subtraction.

### 2.6 Anti-adherence activity of *C. verum* EO against *Candida* spp. strains by the percentage of adherence inhibition (PICA) determination

The effect of *C. verum* EO on microbial adherence to an inert substratum was assessed using the crystal violet microtitration method (Stepanović et al., 2000). Microbial strains were cultured with sub-inhibitory concentrations of the tested solutions (MIC/2 and MIC/4) following the protocol for quantitative antimicrobial evaluation. After incubation, adherent cells were fixed with 99% methanol, stained with 1% crystal violet, resuspended in 33% acetic acid and the determination of the microbial adherence inhibition percentage (MAC%) as previously shown (Corbu et al., 2021).

### 2.7 Determination of the optimal combination of fluconazole and *C. verum* EO

In this study, yeast strains from the genus *Candida* identified and mentioned in Section 2.4 were used.

The microorganisms were stored at −80 °C in a cryoprotective medium and re-cultured on appropriate solid media before each experiment. Microbial suspensions were adjusted to a turbidity equivalent to the 1 McFarland standard (3⨯10^8^ colony forming units, CFU/mL).

The antibiotic used in the study was fluconazole (Sigma-Aldrich). The working solutions were prepared in DMSO and used in the concentration range of 0–32 μg/mL, according to the experimental design (Table 1). The cinnamon EO was dissolved in DMSO. The final concentrations used in the experiments, as shown in Table 1, ranged from 0 to 6.25 μL/mL.

**TABLE 1:** Matrix for the mixture design for EO and fluconazole combination.

No.	Fluconazole concentration (µg/mL)	EOs concentration (µL/mL)
1	11.9	6.3
2	18.2	2.6
3	10.9	4.2
4	12	6.3
5	0.6	6.3
6	32	5.3
7	0	4.1
8	18	0
9	5.9	2.2
10	32	2.7
11	4.5	0
12	4.5	0
13	18.2	2.6
14	32	5.3
15	18.2	2.6
16	32	0

The antimicrobial activity was evaluated using the adapted diffusion method. The solid media were uniformly inoculated with standardized microbial suspensions, using an inoculum adjusted to 1 McFarland. Subsequently, 10 µL from each stock solution mixture according to Table 1 were spotted on the solid medium. The plates were incubated at 37 °C for 24 h. After incubation, the diameters of the inhibition zones (IZD) were measured in millimetres (mm). All determinations were performed in triplicate, and the reported values represent the average of the measurements.

For the selection of the optimal variant, the experimental design was carried out using the Response Surface Methodology (RSM), with an I-optimal design and a quadratic model. Two independent factors were studied: fluconazole concentration (A, 0–32 mg/mL) and EO concentration (B, 0–6.25 μL/mL). An I-optimal design was used, constructed through the coordinate exchange method, to optimise the model prediction efficiency. The design included 16 experiments, randomly distributed without the use of experimental blocks. Mathematical models were generated for each response (the IZD specific to each strain), using linear or quadratic models, depending on statistical significance.

The experimental data were analyzed using analysis of variance (ANOVA) to determine the relevance of the models and individual variables. The threshold for statistical significance was set at *p* < 0.05. The quality of the models was evaluated based on the coefficient of determination (*R* ^2^), adjusted *R* ^2^, predicted *R* ^2^, and the signal-to-noise ratio (Adeq Precision). Lack of fit was used for model validation. Statistical analysis and modelling were performed using Design-Expert software (trial version, version 12, Stat-Ease Inc., USA).

The optimisation was performed using the desirability function, with the aim of identifying an optimal combination between the antibiotic and the EO concentration. The objectives of the optimisation were based on maximising antimicrobial activity (the larger the IZDs, the better) and minimising the concentrations of the tested factors (Table 2). The optimal solution was selected based on the overall desirability value.

**TABLE 2:** Setting objectives, limits, and weights within the desirability function for multi-response optimization.

*Name*	*Goal*	*Lower limit*	*Upper limit*	*Lower weight*	*Upper weight*	*Importance*
A: Fluconazole	Minimize	0	32	1	1	3
B: *C. verum* EO	Minimize	0	6.25	1	1	3
*C. auris* DSM 21092	Maximize	0	41.66	1	1	3
*C. albicans* ATCC 10231	Maximize	0	38.33	1	1	3
*C. parapsilosis* ATCC 22019	Maximize	7	50.66	1	1	3
*C. tropicalis* DSM 7524	Maximize	0	38	1	1	3
*C. auris* 2851	Maximize	0	32.33	1	1	5
*C. auris* 3896	Maximize	0	30.66	1	1	5
*C. auris* 1370	Maximize	0	42	1	1	5
*C. auris* 9069	Maximize	0	28.66	1	1	5
*C. auris* 18,519	Maximize	0	30.33	1	1	5
*C. auris* 6816	Maximize	0	13	1	1	5
*C. auris* 4574	Maximize	0	15.33	1	1	5
*C. auris* 6328	Maximize	0	25	1	1	5

### 2.8 *In vitro* evaluation of the fluconazole - *C. verum* EO combination effects against *Candida* spp.

To evaluate the efficacy of fluconazole (FC) - *C. verum* EO combination, both the reference strain *Candida* spp*.* and a set of clinical isolates were analyzed. The type of pharmacological interaction was determined by calculating the Fractional Inhibitory Concentration Index (FICI), using the following equation:

$$FICI=\frac{MICAcomb}{MICAalone}+\frac{MICBcomb}{MICBalone}$$

where *A* and *B* correspond to the tested agents (EO and FC, respectively). In this context, ​the MIC of each agent tested alone (MICA alone and MICB alone) refers to the minimum inhibitory concentration determined when the compound was used individually. The MIC in combination (MICA comb and MICB comb) represents the concentration of each agent when used together, corresponding to the most effective inhibitory combination.

A value of FICI < 0,5 indicates a synergistic effect, whereas values between 0,5 and 1 suggest an additive interaction. Furthermore, the combination is considered indifferent if the FICI falls between 1 and 2, while any value exceeding 2 signals an antagonistic effect, indicating a reduction in efficacy due to the interaction between the two substances (Gómara and Ramon-Garcia, 2019; Nedea et al., 2025a).

### 2.9 Hemocompatibility

The hemolysis test used ram red blood cells (RBCs) and involved mixing 9 mL of blood with 1 mL of 10% citric acid dextrose to prevent clots. The mixture was centrifuged at 5,000 rpm for 10 min at 4 °C, discarding the platelet-poor plasma and resuspending the RBCs in 10 mL of PBS. Following the methodology outlined by Marinas et al. (2026), a 100 μL aliquot of the stock sample was combined with 400 μL of erythrocyte suspension, incubated at 37 °C for 60 min. Positive and negative controls were created using Triton-X100 and PBS, respectively. After centrifugation, the supernatant was analyzed for absorbance at 540 nm to measure free hemoglobin levels.

### 2.10 *In vivo* evaluation using the *Galleria mellonella* model

#### 2.10.1 *In vivo* evaluation of the antifungal effects of fluconazole and *C. verum* EO against reference *Candida* spp. using the *G. mellonella* model

The mortality rates associated with infections caused by *Candida* spp. isolates were assessed using the *G. mellonella* model. Moreover, the therapeutic potential of the tested compounds was determined, and the *in vivo* expression of fungal virulence factors and fungal viability following treatment. Larvae ranging between 200 and 300 mg were acclimated to 15 °C for 24 h before the experiment. A 10 µL injection (1.0 McFarland) of reference strains (*C. albicans* ATCC 10231, *C. auris* DSM 21092, *C. parapsilosis* ATCC 22019, and *C. tropicalis* DSM 7524) was administered to each of the seven experimental groups into the hemocoel. A control group of uninfected larvae was maintained under identical conditions. To prevent mechanical stress and leakage, therapeutic injections (10 µL) were performed in a different abdominal segment 2 h post-infection. Treatments consisted of FC, *C. verum* EO, or their combination at sub-inhibitory concentrations (MIC/2 and MIC/4). Following the treatment administration, larvae were incubated at 37 °C and monitored at 2, 6, 12, 24, and 48 h post-infection.

#### 2.10.2 Evaluation of *Candida* soluble virulence factors

Following the previously described experimental procedure (2.10.1), the larvae were incubated for 48 h at 37 °C. Subsequently, homogenates were obtained from the surviving larvae. For this purpose, the larvae were homogenized in 1000 µL of sterile PBS, followed by centrifugation for 5 min at 10,000 rpm and the resulting pellets were resuspended in sterile physiological saline (AFS) to achieve a turbidity corresponding to the 1 McFarland standard.

The enzymatic activity of the reference strains, *C. auris* DSM 21092, *C. albicans* ATCC 10231, *C. tropicalis* DSM 7524, and *C. parapsilosis* ATCC 22019 was evaluated using specific culture media to detect the production of hemolysin production, lecithinase and caseinase activity. To this end, aliquots of 10 µL from the obtained suspensions were inoculated in triplicate onto specific culture media were inoculated with treated and untreated microbial suspension followed by the determination of the ratio of the colony diameter (C) to the diameter of the specific inhibition zone occurring around the colony (D) (Corbu et al., 2021):

$$Inhibition\;\left(\%\right)=\frac{D2-C2}{D1-C1}\times 100$$

where C1—colony diameter of control strain, D1—inhibitory effect zone diameter of control strain, C2—colony diameter of treated strain, and D2—inhibitory effect zone diameter of treated strain.

All assays were performed by spot-inoculating the fungal suspension onto the respective media, followed by incubation at 37 °C for 24 h.

The secretion of hemolysins - extracellular toxic lipoproteins that induce hemolysis by forming pores in host cell membranes was detected using SDA supplemented with 5% sheep blood. Following a 24 h incubation period at 37 °C, enzymatic activity was identified by the presence of a characteristic halo around the colonies. Specifically, β- hemolysis was recorded upon the observation of a transparent halo, indicating complete hemoglobin degradation, whereas ɑ-hemolysis was characterized by a greenish halo, reflecting partial hemoglobin degradation (Lazăr et al., 2004).

Lecithinase production was assessed using 2.5% egg yolk supplemented SDA medium, where phospholipid hydrolysis leads to an insoluble precipitate, detected as an opaque halo around the colony, indicating enzyme secretion (Corbu et al., 2024).

Protease activity was assessed on 15% skim milk supplemented SDA medium, where casein degradation leads to the formation of a white-yellowish precipitate zone, indicating protease secretion (Lazăr et al., 2004).

#### 2.10.3 Evaluation of larval mortality

In accordance with the procedure detailed in Section 2.10.1, the larval mortality rate was calculated as follows (Anower et al., 2025):

$$M=\frac{Dt}{Ni}x100,where\;Dt\;is\;the\;number\;of\;dead\;larvae\;and\;Ni\;is\;the\;initial\;population.$$

#### 2.10.4 Evaluation of fungal load

The supernatant obtained from the larval homogenate (Section 2.10.2) was utilized to quantify the microbial load. The fungal load for the reference strains (*C. auris* DSM 21092, *C. albicans* ATCC 10231, *C. tropicalis* DSM 7524, and *C. parapsilosis* ATCC 22019) was subsequently calculated and expressed as CFU/mL as follows:

$CFU/mL=\frac{N\;x\;DF}{V}$ ; where N represents the number of colonies counted on the CHROMagar™ *Candida* Plus plate, DF is the dilution factor (10 for the $10^{-1}$ dilution, established as the optimal threshold for ensuring accurate and reproducible CFU/mL counts), and V denotes the volume of the inoculum plated, expressed in mL.

## 3 Results

### 3.1 Physico-chemical characterisation of *C. verum* EO

The density and refractive index measurements for the *C. verum* EO were consistent with previously reported values found in the literature (Boughendjioua and Djeddi, 2018; Islam et al., 2025; EFSA, 2022) and the PubChem database (National Center for Biotechnology Information, 2025; EFSA Panel on Food Additives and Flavourings, 2022). Specifically, the density of 1.0154 ± 0.0468 g/mL was consistent with the range of 1.010–1.030 at 25 °C for cinnamon bark EO, as reported by PubChem (Table 3). Furthermore, the refractive index of 1.5436 ± 0.0014 was situated between the established limits for cinnamon leaf oil (1.53 - 1.54 at 20 °C) and cinnamon bark oil (1.573 - 1.591 at 25 °C). This concordance serves to confirm its physicochemical characteristics and supports the compositional profile determined through GC-MS analysis.

**TABLE 3:** The physicochemical characteristics of the *C. verum* EO.

Sample	Refractive index	Density (g/mL)
*C. verum* EO	1.5436 ± 0.0014	1.0154 ± 0.0468

The chemical composition of the sample, as determined by GC-MS, is shown in Table 4.

**TABLE 4:** The chemical composition of *C. verum* EO resulted from GC-MS (SD = standard deviation for three measurements).

Class of compounds	Compounds	RI	CAS	Relative content ± Sd (%)
Monoterpenes				
Acyclic				
Alcohols	Linalool	1088	78-70-6	1.10 ± 0.05
	Geraniol	1242	106-24-1	0.40 ± 0.01
Monocyclic				
Hydrocarbon	α-Phellandrene	997	99-83-2	0.06 ± 0.00
	Limonene	1021	138-86-3	0.38 ± 0.02
	γ-Terpinene	1049	99-85-4	0.03 ± 0.00
	α-Terpinolene	1078	586-62-9	0.03 ± 0.00
Alcohols	Terpinen-4-ol	1168	562-74-3	0.05 ± 0.00
	α-Terpineol	1181	98-55-5	0.57 ± 0.02
Aromatics	p-Cymene	1016	99-87-6	0.35 ± 0.02
Bicyclic				
Hydrocarbon	α-Thujene	924	2867-05-2	0.17 ± 0.01
	α-Pinene	931	80-56-8	0.10 ± 0.00
	β-Thujene (Sabinene)	971	3387-41-5	0.12 ± 0.00
Alcohols	Borneol	1159	507-70-0	0.02 ± 0.00
Ethers	Ecualyptol	1024	470-82-6	0.29 ± 0.01
Sesquiterpenes				
Monocyclic				
Hydrocarbon	α-Caryophyllene (Humulene)	1146	6753-98-6	0.34 ± 0.01
Bicyclic				
Hydrocarbon	β-Caryophyllene	1411	87-44-5	2.95 ± 0.14
	β-Selinene	1488	17,066-67-0	0.08 ± 0.00
Epoxide	Caryophyllene oxide	1582	1139-30-6	0.15 ± 0.00
Polycyclic				
Hydrocarbon	α-Copaene	1364	3856-25-5	0.20 ± 0.01
Alcohols	Epi-Cubenol	1621	19,912-67-5	0.03 ± 0.00
Phenylpropanoids				
Phenylpropanoid aldehydes	(Z)-Cinnamaldehyde	1212	57,194-69-1	0.32 ± 0.01
	(E)-Cinnamaldehyde	1281	14,371-10-9	66.50 ± 1.01
Phenylpropanoid ethers	Safrole	1286	94-59-7	0.09 ± 0.00
Phenylpropanoid alcohol	(E)-Cinnamyl alcohol	1301	4407-36-7	0.02 ± 0.00
Phenolic phenylpropanoid	Eugenol	1351	97-53-0	19.14 ± 0.72
Phenylpropanoid esters	Cinnamyl acetate	1431	103-54-8	3.82 ± 0.21
	Eugenol acetate	1510	93-28-7	1.23 ± 0.06
Phenylpropanoid carboxylic acids	(E)-Cinnamic acid	1437	140-10-3	0.25 ± 0.01
Total monoterpene hydrocarbons				1.24 ± 0.05
Total oxygenated monoterpenes				2.43 ± 0.09
Total sesquiterpene hydrocarbons				3.57 ± 0.16
Total oxygenated sesquiterpenes				0.18 ± 0.00
Total phenylpropanoids				91.37 ± 2.02
Total identified compounds				98.79 ± 2.32

GC-MS analysis of *C. verum* EO showed that phenylpropanoids were the major class (91.37%). The main compound was (E)-cinnamaldehyde (66.50%) followed by eugenol (19.14%). Other identified compounds were cinnamyl acetate (3.82%), eugenol acetate (1.23%) (E)-cinnamic acid (0.25%), safrole (0.09%) and (E)-cinnamyl alcohol (0.02%). Monoterpenes constituted 3.67% of the composition, with linalool (1.10%), α-terpineol (0.57%), limonene (0.38%) and p-cymene (0.35%) as the main components. Sesquiterpenes represented 3.75%, the most abundant representative of this class was β- caryophyllene (2.95%).

### 3.2 Evaluation of the chemical stability of fluconazole in the presence of *C. verum* EO through HPLC

**FIGURE 1:**
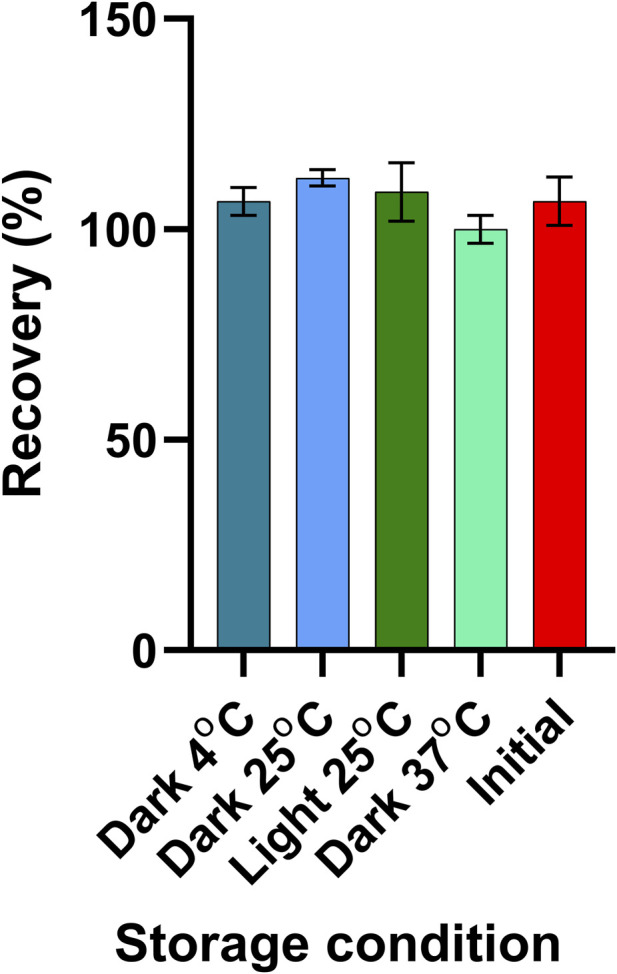
Evaluation of the fluconazole recovery percentage based on storage conditions through one-way ANOVA analysis (n = 3) followed by the Tukey test for multiple comparisons, with no significant differences compared to the initial sample (*p* > 0.05).

The chemical stability of fluconazole in the presence of *C. verum* EO was evaluated by HPLC after 30 days under different storage conditions (dark at 4 °C, 25 °C, and 37 °C, light at 25 °C) (Figure 1). The recovery values, which ranged from 100% to 112.22%, showed that there was no significant chemical degradation. The highest recovery was at 25 °C in the dark (112.22% ± 1.92%), and the lowest was at 37 °C in the dark (100% ± 3.33%), which suggests that stability may have decreased slightly with temperature. However, all values were above 90%, which confirms that fluconazole is chemically compatible with *C. verum* EO and supports the use of this combination in further experiments. The detailed data can be found in the Supplementary Material (Supplementary Table S1).

The one-way ANOVA test (Figure 1) showed that there are no statistically significant differences between the stored samples and the initial sample (*p* > 0.05). This result confirms that neither temperature nor light exposure significantly influenced the recovery of fluconazole under the investigated experimental conditions. The treated fluconazole showed good chemical stability in all the conditions, preserving its integrity and recovery within acceptable limits.

### 3.3 *Candida* spp. strains characterization

E-test MIC determination showed that the 8 *C. auris* isolates were non-susceptible to fluconazole and amphotericin B, whereas susceptibility was observed for micafungin, caspofungin, and flucytosine as previously shown (Nedea et al., 2025b).

### 3.4 Antifungal efficacy of *C. verum* EO

**FIGURE 2:**
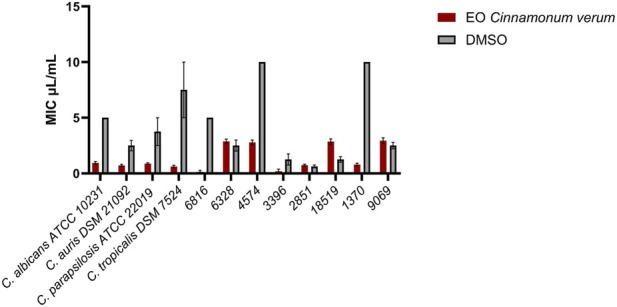
Average MIC values of *C. verum* EO and DMSO against tested *Candida* strains.

The MIC values of *C. verum* EO were determined and compared with the solvent control (DMSO), revealing a significant antifungal activity against all tested strains, indicating a broad-spectrum antifungal effect (Figure 2, Supplementary Table S2). The average MIC values obtained for *C. verum* EO were notably lower than those recorded for the DMSO control. The obtained MIC values for *C. verum* EO ranged from 0.14 to 2.94 μL/mL. Among the reference strains, the EO exhibited notable activity, with MIC values of 0.62 ± 0.11 μL/mL for *C. tropicalis*, 0.72 ± 0.10 μL/mL for *C. auris*, 0.88 ± 0.08 μL/mL for *C. parapsilosis*, and 0.95 ± 0.12 μL/mL for *C. albicans*. Regarding the clinical isolates, the highest susceptibility was observed for *C. auris* 6816 (0.14 ± 0.14 μL/mL) and 3396 (0.19 ± 0.19 μL/mL), while the lowest susceptibility was recorded for *C. auris 9069* (2.94 ± 0.27 μL/mL), 6328 (2.88 ± 0.20 μL/mL), 18,519 (2.85 ± 0.25 μL/mL), and 4574 (2.78 ± 0.23 μL/mL) strains. In comparison, the solvent control (DMSO) showed significantly higher MIC values, ranging from 0.63 to 10 μL/mL across the tested strains. For reference strains, DMSO MIC values were 2.5–7.5 μL/mL, whereas for clinical isolates they reached up to 10 μL/mL.

### 3.5 Anti-adherence efficacy of *C. verum* EO

**FIGURE 3:**
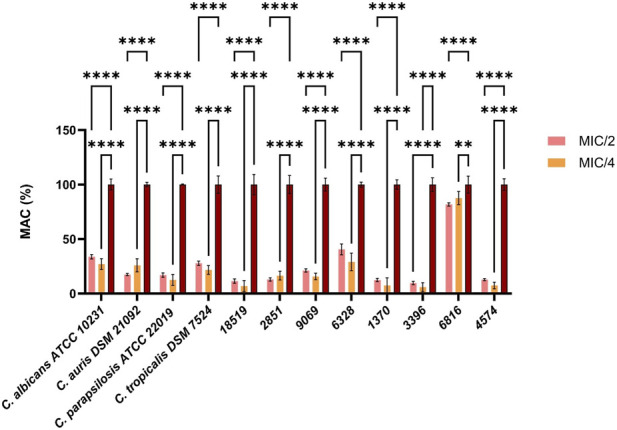
Microbial adherence percentage (MAC%) values for *C. verum* EO compared to the control *Candida* strains (** *p* < 0,01, **** *p* < 0,0001) (Dunnett’s multiple comparisons test).

The influence of *C. verum* EO on the adherence capacity of *Candida* strains to inert substratum was evaluated at sub-inhibitory concentrations (MIC/2 and MIC/4). The obtained results, shown in Figure 3 and Supplementary Table S3, indicate a significant inhibition of PICA% at both tested concentrations compared to the control. At MIC/2, strong inhibitory effects were observed for most strains, with PICA% values exceeding 70%. Notably, *C. auris* clinical isolates such as 3396 (90.36%), 18,519 (88.51%), 2851 (87.00%), and 1370 (87.58%) exhibited high susceptibility, indicating a significant reduction in their adherence ability to inert substrates. Among the reference strains, the inhibition of adherence capacity decreases in the following order: C*. parapsilosis* ATCC 22019 > *C. auris* DSM 21092 > *C. albicans* ATCC 10231 (83%/ 82.5%/ 66.20%). At MIC/4 sub-inhibitory concentration the highest PICA% corresponded in decreasing order to 3396 (94.09%), 18,519 (93.06%), and 1370 (92.56%). *Candida parapsilosis* ATCC 22019 (87.41%) and *C. tropicalis* DSM 7524 (78.37%) and opposite the lower PICA% corresponded to one 6816 (12.32%). In the case of *C. auris* clinical strains (18,519, 1370, 3396, and 4574) it was obtained higher PICA% values at MIC/4 comparatively to MIC/2 demonstrating the efficacy of this concentration against adherence ability.

### 3.6 Evaluation of the optimal combination between fluconazole and *C. verum* EO

Based on the results obtained in subchapter 3.4 and considering the MIC value recommended by the CLSI standard for fluconazole (32 μg/mL), the antifungal effect of 16 possible combinations of the two components was qualitatively evaluated (Table 5).

**TABLE 5:** Experimental design matrix with the experimental values for IZD (mm) against all yeast strains studied using antifungal agent and EO mixture.

*No*	*Concentration fluconazole (µg/mL)*	*Concentration EO (µL/mL)*	*IZD (mm)*											
			*C. auris DSM 21092*	*C. albicans ATCC* *10,231*	*C. parapsilosis ATCC* *22,019*	*C. tropicalis DSM 7524*	*C. auris 2851*	*C. auris 3896*	*C. auris 1370*	*C. auris 9069*	*C. auris 18,519*	*C. auris 6816*	*C. auris 4574*	*C. auris 6328*
*1*	11.9	6.25	19	30.33	41	35.33	32.33	30.66	17.33	21	25	12.66	9.33	8
*2*	18.2	2.6	31.33	21	39.66	38	8	8.33	8.66	0	12	7.66	8.66	10.66
*3*	10.9	4.2	30.66	32.66	38.33	28	20.66	27.33	15	21.33	25.33	7	10	6.66
*4*	12	6.25	15	38.33	35	30.33	17.33	24	25	15	30.33	11.33	13	11
*5*	0.6	6.25	21	21.33	7	10	7.66	16	20	15.33	20	7.33	5.33	15
*6*	32	5.31	41.66	0	45	3.33	1	0	0	0	0	11	10.33	15.33
*7*	0	4.06	15	5	8.33	0	13.33	5	12.33	7	12.33	5.66	4	5
*8*	18	0	21	0	40.66	0	0	0	0	0	0	0	0	0
*9*	5.9	2.19	10.33	25.66	31.33	10	13.33	22.66	5	22	7	0	0	4.33
*10*	32	2.66	21	30	50	12.33	17	26	0	7.33	17.66	0	0	0
*11*	4.5	0	0	0	31.33	0	0	0	15.33	0	0	0	0	0
*12*	4.5	0	0	0	30	0	0	0	0	0	0	0	0	0
*13*	18.2	2.63	20.66	31.33	50.66	30	32	28.33	42	15	20.33	13	15.33	25
*14*	32	5.31	24.66	30	41.33	25.66	15	27	30.66	15	11.33	0	0	0
*15*	18.2	2.63	15	30.66	36	26.33	29	27.33	30	28.66	20.33	0	5	0
*16*	32	0	25	0	40	0	0	0	0	0	0	0	0	0

#### 3.6.1 Analysis of variance and model optimization for the mixture of EOs - fluconazole against each strain under study

The comparison of regression models (linear, two-factor interaction and quadratic models) for the 12 response variables indicated that the quadratic models were the best fit for most of the responses analysed (Table 6). The responses related to *Candida* spp. Strains (R1–R4) showed high values of the coefficient of determination (*R* ^2^), particularly for the quadratic models (e.g., R3: *R* ^2^ = 0.8723; R4: *R* ^2^ = 0.8007). Most models had Adeq Precision values greater than 4, which suggests an adequate signal-to-noise ratio to be used in the optimisation process.

**TABLE 6:** Regression analysis summary for chosen models’ fitting data for each response.

*Model*	*R* ^*2*^	*Adjusted R* ^*2*^	*Adeq precision*	*SD*	*Mean*	*% CV*	*p-Value*	*Lack of fit (F-value)*
*R1: C. auris* DSM 21092								
*Linear*	*0.5673*	*0.5007*	*8.1629*	*7.58*	*19.46*	*38.96*	*0.0043*	*1.10*
*Quadratic*	*0.6370*	*0.4555*	*5.7786*	*7.92*	*19.46*	*40.48*	*0.0432*	*1.24*
*2FI*	*0.5994*	*0.4993*	*6.9873*	*7.59*	*19.46*	*39.02*	*0.0098*	*1.10*
*R2: C. albicans* ATCC 10231								
*Quadratic*	*0.6692*	*0.5038*	*5.4994*	*10.40*	*18.52*	*56.16*	*0.0287*	*0.60*
*Linear*	*0.3412*	*0.2399*	*4.2367*	*12.87*	*18.52*	*69.51*	*0.0664*	*1.14*
*2FI*	*0.3480*	*0.1850*	*3.9055*	*13.33*	*18.52*	*71.98*	*0.1491*	*1.25*
*R3: C. parapsilosis* ATCC 22019								
*Quadratic*	*0.8723*	*0.8084*	*11.0407*	*5.40*	*35.35*	*15.27*	*0.0003*	*0.66*
*Linear*	*0.5928*	*0.5301*	*8.3499*	*8.46*	*35.35*	*23.92*	*0.0029*	*2.12*
*2FI*	*0,6389*	*0.5486*	*8.1754*	*8.29*	*35.35*	*23.44*	*0.0054*	*2.05*
*R4: C. tropicalis* DSM 7524								
*Quadratic*	*0.8007*	*0.7010*	*7.3659*	*7.89*	*15.58*	*50.66*	*0.0028*	*0.61*
*Linear*	*0.2835*	*0.1733*	*3.7823*	*13.13*	*15.58*	*84.24*	*0,1145*	*2.32*
*2FI*	*0.2835*	*0.1044*	*3.1478*	*13.66*	*25.58*	*87.68*	*0.2449*	*2.58*
*R5: C. auris 2851*								
*Quadratic*	*0.6338*	*0.4507*	*5.0759*	*8.48*	*12.91*	*65.63*	*0.0448*	*2.58*
*Linear*	*0.2234*	*0.1040*	*3.1036*	*10.83*	*12.91*	*83.82*	*0.1933*	*4.16*
*2FI*	*0.2299*	*0.0374*	*3.0310*	*11.22*	*12.91*	*86.88*	*0.3535*	*4.58*
*R6: C. auris 3896*								
*Quadratic*	*0.5765*	*0.3647*	*4.5066*	*10.09*	*15.16*	*66.50*	*0.0834*	*0.77*
*Linear*	*0.3079*	*0.2014*	*3.9479*	*11.31*	*15.16*	*74.56*	*0.0914*	*1.07*
*2FI*	*0.3102*	*0.1377*	*3.4417*	*11.75*	*15.16*	*77.48*	*0.2010*	*1.18*
*R7: C. auris 1370*								
*Linear*	*0.1837*	*0.0581*	*3.2792*	*12.73*	*13.83*	*92.02*	*0.2673*	*0.6581*
*Quadratic*	*0.3322*	*−0.0017*	*3.3341*	*13.13*	*13.83*	*94.90*	*0.4677*	*0.6912*
*2FI*	*0.1880*	*−0.0149*	*2.8291*	*13.21*	*13.83*	*95.53*	*0.4577*	*0.7255*
*R8: C. auris 9069*								
*Quadratic*	*0.5120*	*0.2681*	*4.1739*	*8.43*	*10.48*	*80.48*	*0.1491*	*1.05*
*Linear*	*0.2831*	*0.1728*	*4.6132*	*8.97*	*10.48*	*85.56*	*0.1149*	*1.22*
*2FI*	*0.2898*	*0.1122*	*3.7325*	*9.29*	*10.48*	*88.64*	*0.2341*	*1.34*
*R9: C. auris 18,519*								
*Quadratic*	*0.7926*	*0.6889*	*7.9659*	*5.86*	*12.60*	*46.52*	*0.0034*	*1.87*
*Linear*	*0.4933*	*0.4154*	*6.7092*	*8,04*	*12.60*	*63.77*	*0.0120*	*3.62*
*2FI*	*0.5626*	*0.4533*	*7.0486*	*7.77*	*12.60*	*61.67*	*0.0162*	*3.43*
*R10: C. auris 6816*								
*Linear*	*0.4509*	*0.3665*	*5.8325*	*4.18*	*4.73*	*88.51*	*0.0203*	*0.17*
*Quadratic*	*0.5726*	*0.3589*	*5.0313*	*4.21*	*4.73*	*89.03*	*0.0867*	*0.0950*
*2FI*	*0.4553*	*0.3191*	*4.8169*	*4.34*	*4.73*	*91.76*	*0.0558*	*0.1857*
*R11: C. auris 4574*								
*Quadratic*	*0.6177*	*0.4266*	*5.3272*	*4.07*	*5.06*	*80.32*	*0.0540*	*0.24*
*Linear*	*0.3250*	*0.2211*	*4.1410*	*4.74*	*5.06*	*93.61*	*0.0777*	*0.5204*
*2FI*	*0.3263*	*0.1579*	*3.5550*	*4.93*	*5.06*	*97.34*	*0.1774*	*0.5764*
*R12: C. auris 6328*								
*Linear*	*0.2431*	*0.1267*	*3.7148*	*6.97*	*6.31*	*110.44*	*0.1636*	*0.14*
*Quadratic*	*0.3212*	*−0.0182*	*2.7147*	*7.53*	*6.31*	*119.25*	*0.4924*	*0.1360*
*2FI*	*0.2491*	*0.0614*	*3.1751*	*7.23*	*6.31*	*114.49*	*0.3114*	*0.1524*

For responses R5–R12, lower values of *R* ^2^ and adjusted *R* ^2^ were observed, associated with higher values of the coefficient of variation (%CV). Most of the quadratic models were statistically significant (p < 0.05), especially for *C. auris* DSM 21092, *C. albicans* ATCC 10231, *C. parapsilosis* ATCC 22019, *C. tropicalis* DSM 7524, and *C. auris* 18,519. The selected variants for final optimisation are presented in Table 6. Some responses, especially R6–R8 and R11–12, were not statistically significant. The low F values obtained from the lack-of-fit tests showed that the models fit well with the experimental data.

Diagnostic analysis of the regression models showed that most of the studentized residuals were within the ±3 range indicating good agreement between the observed and the predicted values. Although some experimental points showed high values for Cook’s distance and DFFITS, these were numerically limited and did not significantly affect the robustness of the models.

The average values of the studentized residuals varied between 0.76 and 0.90 for all the analysed strains. The RMSE values were relatively similar between strains, ranging from 1.00 to 1.35. The percentage of external standardised residues >2 ranged from 0% to 12.5%, with the best adjustments for *C. auris* DSM 21092 and *C. parapsilosis* ATCC 22019**.**

Average values of the influence indicators showed low values of Cook’s distance (<0.15 for most strains), and DFFITS values were generally below the critical thresholds (Table 7). Overall, the statistical and diagnostic analysis confirmed the adequacy of the developed models for describing the interaction effects between fluconazole and EO on the MIC values for different strains of *Candida* spp. The individual data and corresponding diagnostic plots are presented in the Supplementary Material (Supplementary Tables S4–S15 and Supplementary Figure S1).

**TABLE 7:** Global diagnostic indicators of regression models for IZD against *Candida* spp. Strains.

Microbial strain	Mean of internally studentized residuals	RMSE	% Problematic observations	Mean of Cook’s distance	Mean of influence on fitted value DFFITS
*C. auris* DSM 21092	0.9006	1.0241	0	0.0741	0.424
*C. albicans* ATCC 10231	0.8777	1.2708	6.25	1.2708	0.7174
*C. parapsilosis* ATCC 22019	0.8245	1.0079	0	0.0736	0.5804
*C. tropicalis* DSM 7524	0.8664	1.2017	12.5	0.1306	0.7288
*C. auris* 2851	0.8346	1.0455	6.25	0.0802	0.6113
*C. auris* 3896	0.8407	1.1505	6.25	0.1045	0.6448
*C. auris* 1370	0.8277	1.1091	6.25	0.0603	0.3661
*C. auris* 9069	0.7644	1.0705	6.25	0.0792	0.5611
*C. auris* 18,519	0.8734	1.1039	12.5	0.1009	0.668
*C. auris* 6816	0.7977	1.1053	12.5	0.0657	0.3506
*C. auris* 4574	0.8093	1.1206	12.5	0.0637	0.3569
*C. auris* 6328	0.7908	1.3528	6.25	0.0500	0.3244

#### 3.6.2 The influences of fluconazole and EO on the antifungal effect

The analysis of the perturbation diagrams showed that fluconazole (factor B) had the greatest influence on the MIC values for most of the analysed strains, as evidenced by the steeper slopes of the associated curves (Supplementary Figure S1). For *C. auris* DSM 21092, *C. parapsilosis* ATCC 22019, and *C. tropicalis* DSM 7524, the influence of fluconazole was lower, while *C. verum* EO (factor A) exhibited a comparable or more pronounced effect.

#### 3.6.3 3D response surface plots

The response surfaces obtained using the RSM method exhibited a nonlinear relationship between the examined variables and the IZD (Supplementary Figure S2). Most models exhibited convex shapes, indicating the existence of optimal levels of fluconazole-EO pairs. Fluconazole typically exerted a more pronounced effect on the observed response, as evidenced by steeper surface slopes along the pertinent axis. In contrast, *C. verum* EO demonstrated a dose-dependent response that often aligned with a quadratic model, implying optimal efficacy at intermediate doses, with a possibility of attenuation at higher doses. Notable differences were observed among various strains, particularly *C. auris*, *C. parapsilosis*, and *C. tropicalis*.

Fluconazole’s antifungal effectiveness was reduced, while the impact of EO was increased. This observation suggests that different antifungal response mechanisms were involved. These results corroborate the hypothesis that *C. verum* EO may augment the potency of fluconazole, especially against strains exhibiting reduced susceptibility to azole antifungals.

#### 3.6.4 Multi-response optimisation through the desirability function based on imposed constraints

The desirability function allowed the identification of an optimal combination of factors, corresponding to a fluconazole concentration of 15.00 μg/mL and an EO concentration of *C. verum* of 5.00 μL/mL (Table 8).

**TABLE 8:** The optimal solution obtained through the utility function: factor levels and estimated responses.

Parameter	Fluconazole	*C. verum* EO	C. Auris DSM 21092	*C. albicans* ATCC 10231	*C. Parapsilosi*s ATCC 22019	*C. Tropicali*s DSM 7524	*C. auris* 2851	*C. auris* 3896	*C. auris* 1370	*C. auris* 9069	*C. auris* 18,519	*C. auris* 6816	*C. auris* 4574	*C. auris* 6328	Desirability
Predicted	14.81	5.11	22.93	34.03	40.1	33.63	24.44	27.16	18.52	19.03	24.02	7.70	11.00	9.42	**0.609** **Selected**
Experimental	15.00	5.00	20.33 ± 1.25	35.33 ± 0.47	41 ± 1.41	36.33 ± 0.47	21.33 ± 0.94	29 ± 0.82	16.33 ± 0.47	20.33 ± 0.94	26 ± 0.82	6.33 ± 0.47	12.33 ± 0.47	9.33 ± 0.94	​
Absolute difference			−2.60	1.30	0.90	2.70	−3.11	1.84	−2.19	1.30	1.98	−1.37	1.33	−0.09	​
Percentage deviation			11.34	3.82	2.24	8.03	12.73	6.77	11.83	6.83	8.24	17.79	12.09	0.96	​
The exp/pred ratio			0.89	1.04	1.02	1.08	0.87	1.07	0.88	1.07	1.08	0.82	1.12	0.99	​
Global MAE															**1.726**
RMSE															**1.908**

Bold values indicate the optimal factor levels and the corresponding predicted and experimental responses selected by the desirability function.

The comparison of the model-predicted values with the experimentally obtained ones showed a good overall agreement, with differences being under 10% for most of the analysed responses. For certain strains, including *C. albicans*, *C. tropicalis*, and *C. parapsilosis*, the experimental values were slightly higher than the predicted ones, while for *C. auris* reference and some of the clinical strains (1370, 2851, 6816, and 6328), the experimental values were lower.

**FIGURE 4:**
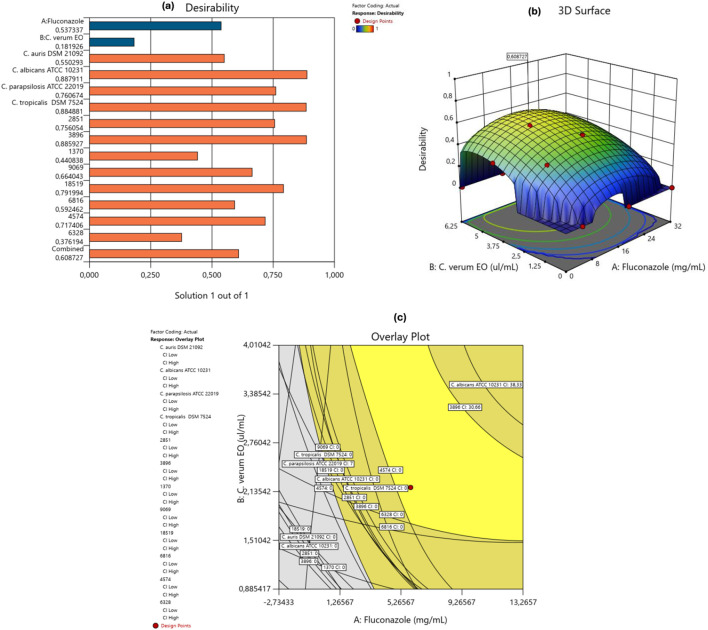
Graphical depiction of multi-response optimization: **(a)** individual desirability function values, **(b)** desirability response surface, and **(c)** optimal domain as determined by the overlay diagram.

Model predictions were in good agreement with the experimental data with MAE of 1.726 mm and RMSE of 1.908 mm, and the small difference between the two values confirmed the relatively uniform distribution of errors. The value of the global desire function was 0.609. The analysis of individual desire functions showed the differences in the analysed responses, with some strains having higher desire values than others (Figure 4a). The response surface of the desirability function showed a global optimum at reduced concentrations of fluconazole and EO, with a maximum value of the desirability function of approximately 0.61 (Figure 4b). The overlay diagram (Figure 4c) showed the operational region where all responses simultaneously satisfied the established acceptability criteria.

### 3.7 Effects of fluconazole - *C. verum* EO combination against *Candida* spp.

The FICI index was determined to assess the *in vitro* interaction between fluconazole and *C. verum* EO against *Candida* spp. Strains, and the obtained results are summarized in Table 9. The calculated FICI values indicate that the combination of *C. verum* EO with fluconazole produced mainly synergistic interactions against the tested *Candida* strains, including MDR clinical isolates of *C. auris* [7 out of 8 clinical strains showed synergy (FICI ≤ 0.5), with values ranging from 0.04 to 0.47, indicating consistent potentiation of fluconazole activity by *C. verum* EO. In one strain (2851) it was obtained with additive effect (FICI = 0.65) (Table 9).

**TABLE 9:** FICI values for the combination of fluconazole and *C. verum* EO against *Candida* spp. strains.

Strain	MIC EO	MIC a	FICIa	MIC fluconazole	MIC b	FICIb	FICI	Interpretation
*C. albicans* ATCC 10213	1,88	0,05	0,03	4,00	0,02	0,01	0,03	Synergistic
*C. auris* DSM 21092	0,59	0,03	0,05	32,00	0,02	0,00	0,05	Synergistic
*C. tropicalis* DSM 7524	0,24	0,05	0,21	3,00	0,02	0,01	0,22	Synergistic
*C. prapsilosis* ATCC 22019	0,78	0,05	0,06	3,00	0,02	0,01	0,07	Synergistic
*C. auris* 1370	0,20	0,09	0,46	8,00	0,04	0,01	0,47	Synergistic
*C. auris* 2851	1,56	1,00	0,64	3,00	0,04	0,01	0,65	Additive
*C. auris* 3396	1,56	0,09	0,06	2,00	0,04	0,02	0,08	Synergistic
*C. auris* 4574	0,40	0,09	0,23	8,00	0,04	0,01	0,23	Synergistic
*C. auris* 6328	0,20	0,09	0,46	17,00	0,04	0,00	0,46	Synergistic
*C. auris* 6816	0,73	0,09	0,12	2,00	0,04	0,02	0,14	Synergistic
*C. auris* 9069	0,39	0,09	0,23	3,00	0,04	0,01	0,24	Synergistic
*C. auris* 18,519	3,13	0,09	0,03	5,00	0,04	0,01	0,04	Synergistic

Legend: MICa = MIC, of C. verum EO, in combination; MICb = MIC, of fluconazole in combination; EO = C. verum EO.

Importantly, all fluconazole-resistant strains exhibited very low FICI values (0.05–0.23), suggesting that the presence of cinnamon EO may enhance the antifungal activity of fluconazole and potentially restore antifungal susceptibility in resistant strains.

### 3.8 Hemocompatibility

**FIGURE 5:**
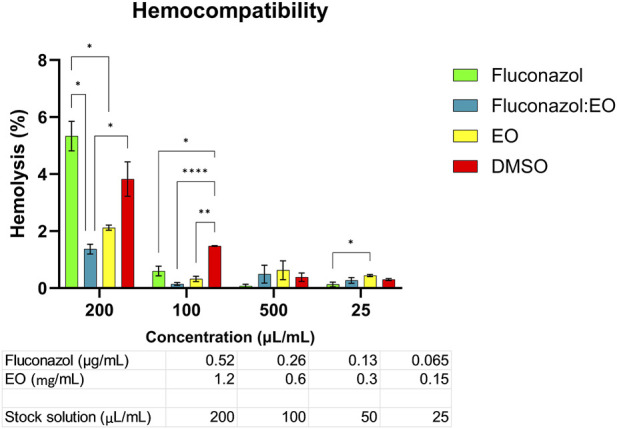
Evaluation of the hemocompatibility of the samples (Fluconazole, Fluconazole:EO, EO, and DMSO) at different concentrations, expressed as a percentage of haemolysis (%). The statistical analysis was performed using two-way ANOVA with Geisser–Greenhouse correction, followed by Tukey’s *post hoc* test for multiple comparisons. The symbols for statistical differences are indicated as follows: *p < 0.05, **p < 0.01, ***p < 0.001, ****p < 0.0001.

Figure 5 shows the percentage of erythrocyte haemolysis for fluconazole, the combination Fluconazole: EO, EO and DMSO at concentrations of 200, 100, 50 and 25 μg/mL. All samples showed a concentration dependent effect with the values of haemolysis decreasing as the concentration was reduced. At a concentration of 200 μg/mL, fluconazole showed the highest haemolysis (5.33% ± 0.52%) followed by DMSO (3.83% ± 0.61%), while the Fluconazole:EO combination showed the lowest haemolysis (1.37% ± 0.17%). Therefore, Fluconazole:EO combination has a statistically significant improved hemocompatibility compared to fluconazole alone (*p* < 0.05).

#### 3.8.1 The reduction of hemolysis values in all samples was observed for concentrations of 100 μg/mL, with statistically significant differences with respect to the solvent control

The concentrations of 50 μg/mL and 25 μg/mL showed little or absent hemolysis (< 1%).

### 3.9 Antifungal effects of fluconazole and *C. verum* EO in *G*. *mellonella* infection model

#### 3.9.1 Evaluation of enzymatic virulence factors in *Candida* strains recovered from infected *G. mellonella*

Hemolysin, caseinase, and lipase modulation were selected for further analysis among the virulence factors assessed in the *G. mellonella* model, due to their well-recognized role in fungal pathogenicity and their confirmed expression in reference *Candida* strains (Nedea et al., 2025b). Moreeover, after antifungal treatment administration, these enzymes remained the only virulence factors consistently expressed by the investigated strains.

**FIGURE 6:**
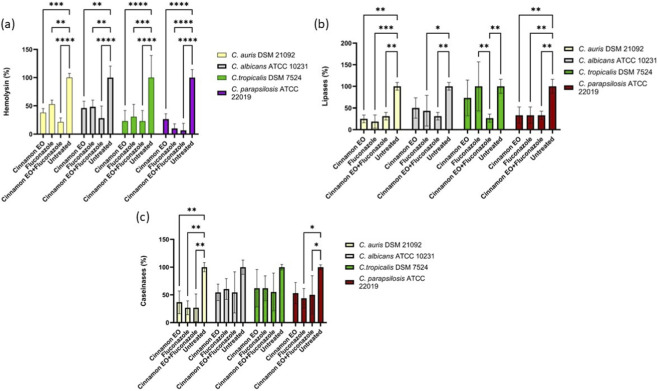
Evaluation of the ability of selected strains to secrete hemolysins **(a)**, lipases **(b)**, or caseinases **(c)** in the presence of EO, fluconazole, and their synergistic combination, or in the absence of treatment. Legend: EO – C. verum EO; FC – fluconazole; None – untreated (**** p < 0.0001, *** p < 0.001, ** p < 0.01, * p < 0.05, ns – not statistically significant).

Figure 6 shows the modulation of virulence factor production in the selected reference strains recovered from the infected *G. mellonella* model, specifically hemolysin (Figure 6a), caseinase (Figure 6b), and lipase (Figure 6c) activities, under conditions of antifungal treatment compared with untreated controls.

Regarding hemolysin secretion (Figure 6a), all treatments significantly reduced hemolysin production compared with the untreated control (100%) across all *Candida* species. Cinnamon EO has significantly reduced hemolysin expression in *C. tropicalis* (76.92% reduction), followed by *C. parapsilosis* (73.33% reduction), *C. auris* (61.95%) and *C. albicans* (53.67%) (*p* < 0.01–0.001). Fluconazole reduced hemolysin in *C. parapsilosis* (90%), *C. tropicalis* (69.23%), *C. albicans* (51.67%) and in *C. auris* (47.37%) (*p* < 0.01–0.0001). The combination of cinnamon EO and fluconazole induced the greatest inhibition, lowering hemolysin by 93.33% in *C. parapsilosis*, followed by *C. auris* (78.58% reduction), *C. tropicalis* (76.92%) and *C. albicans* (71.67%) (*p* < 0.001–0.0001).

For lipase production, the highest efficacy corresponded to the combination of *C. verum* EO with fluconazole in the following decreasing order: *C. tropicalis* (73.33% reduction) > *C. auris* and *C. albicans* (68.75% reduction) > *C. parapsilosis* (66.67% reduction) (*p* < 0.01–0.001). In the case of *C. verum* EO the efficacy was *C. auris* (75%) > *C. parapsilosis* (66.67%) > *C. albicans* (50%) > *C. tropicalis* (26.67%) (*p* < 0.05 - 0.01). Fluconazole that exhibited the lowest inhibitory effect on lipase secretion: *C. auris* (81.25%) > *C. parapsilosis* (66.67%) > *C. albicans* (56.25%) > *C. tropicalis* (0%) (*p* < 0.05 - 0.01) (Figure 6b; Supplementary Table S17S).

Regarding caseinase production fluconazole exhibited the strongest inhibitory effect in *C. auris* (73.33% reduction), followed by *C. parapsilosis* (56.25%), *C. albicans* (39.39%) and *C. tropicalis* (37.93%) (*p* < 0.05 - 0.01). The combination of cinnamon EO and fluconazole produced a higher inhibition in *C. auris* (73.33% reduction) and moderate, comparable reductions in *C. albicans* (45.45%), *C. tropicalis* (44.83%), and *C. parapsilosis* (50.00% reduction) (*p* < 0.05 - 0.01). Cinnamon EO alone demonstrated comparatively lower inhibitory activity, reducing caseinase production in *C. auris* (63.33%), *C. parapsilosis* (46.87% reduction), *C. albicans* (45.45%) and *C. tropicalis* (37.93%) (*p* < 0.01) (Figure 6c; Supplementary Table S18).

#### 3.9.2 Evaluation of larval mortality

**FIGURE 7:**
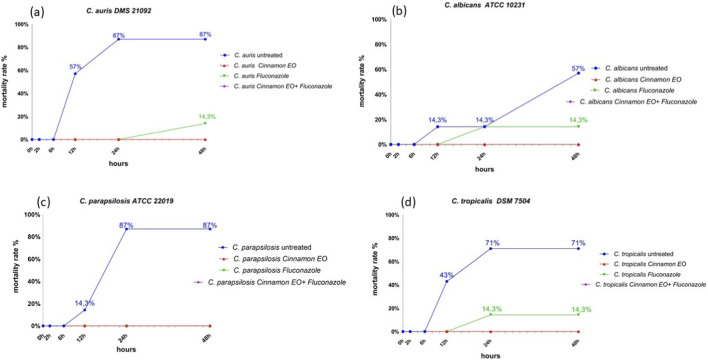
Evaluation of the mortality rate of Galleria mellonella larvae infected with Candida strains in the presence of treatment with cinnamon EO, fluconazole, or cinnamon EO + fluconazole. Panels represent: **(a)** *C. auris* DSM 21092, **(b)** *C. albicans* ATCC 10231, **(c)** *C. parapsilosis* ATCC 22019, and **(d)** *C. tropicalis* DSM 7504.

In all reference strains it was revealed that combination therapy based on *C. verum* EO and fluconazole produced the lowest mortality rates (0 at 48 h post-infection). Fluconazole monotherapy showed limited protective effects, with mortality rates ranging from 0% to 14.3% depending on the inoculated *Candida* species, indicating partial inhibition of fungal proliferation. Specifically, for the *C. auris* strain, the mortality rate at 48 h post-infection was 14.3%; for *C. albicans* and *C. tropicalis*, mortality reached 14.3% at 24 and 48 h, respectively; while for *C. parapsilosis*, no mortality (0%) was observed. Regarding *C. verum* EO, the absence of larval mortality under EO monotherapy exposure was demonstrated. The findings suggest that, although cinnamon EO has been shown to exert anti-virulence effects such as reducing hemolysin and lipase activity, its use alone or alongside other agents does not lead to effective fungal eradication under the tested conditions. Across all species, the treated groups consistently showed lower mortality compared to the untreated controls (*C. auris* and *C. parapsilosis*: 87% at 24–48 h; *C. tropicalis*: 71%; and *C. albicans*: 57%) an unexpected result suggesting that these interventions may not exert a fungicidal effect and could even promote survival or adaptive stress responses (Figure 7).

#### 3.9.3 Quantitative analysis of fungal viability

**FIGURE 8:**
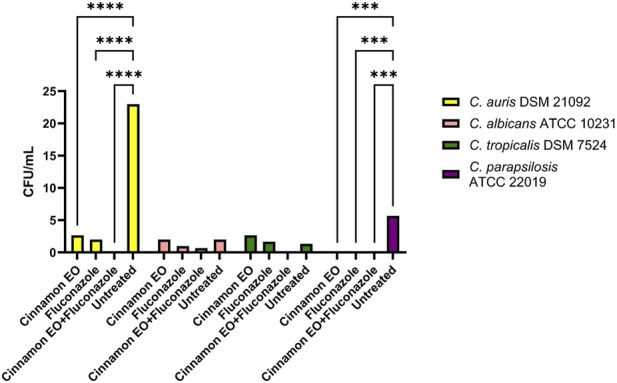
The number of CFU/mL recovered from *G. mellonella* homogenates following the administration of EO, fluconazole and their combination (*** p < 0.001; **** p < 0.0001, ns- not statistically significant).

The microbial load in infected larvae was quantitatively assessed (CFU/mL) after treatment with *C. verum* EO, fluconazole, and respectively with their combination. As illustrated in Figure 8 and Supplementary Table S19, all tested treatments led to a significant decrease in fungal load compared to the untreated control across all *Candida* species [*C. auris* (23 ± 5.72 CFU/mL) > *C. parapsilosis* (5.67 ± 0.94 CFU/mL) > *C. albicans* (2.00 ± 1.41 CFU/mL) > *C. tropicalis* (1.33 ± 0.94 CFU/mL)]. The highest reduction of fungal load corresponds to the combination therapy (*C. verum* + fluconazole) against *C. auris*, *C. tropicalis*, and *C. parapsilosis* isolates 0 CFU/mL), followed by *C. albicans* strain (0.67 ± 0.94 CFU/mL; *p* < 0.001–0.0001 vs. control). In decreasing order Cinnamon EO reduce the microbial growth as follows: *C. auris* and *C. tropicalis* (2.67 ± 2.05 CFU/mL) > *C. albicans* (2.00 ± 0.00 CFU/mL) > *C. parapsilosis* (0 CFU/mL) (*p* < 0.01 - 0.001). Regarding fluconazole, the fungal load was reduced in the following decreasing order: *C. auris* (2.00 ± 0.82 CFU/mL) > *C. tropicalis* (1.67 ± 0.47 CFU/mL) > *C. albicans* (1.00 ± 0.82 CFU/mL) > *C. parapsilosis* (0 CFU/mL) (*p* < 0.01- 0.001).

## 4 Discussion

The study investigates the antifungal efficacy of *C. verum* EO, alone or in combination with fluconazole, against both reference strains and clinical MDR *Candida* isolates, while also examining possible synergistic interactions using a combination of *in vitro* assays and the *G. mellonella in vivo* model.

The *C. verum* EO had a density of 1.0154 ± 0.0468 g/mL and a refractive index of 1.5436 ± 0.0014. These values are in accordance with what has already been published (National Center for Biotechnology Information, 2025; Boughendjioua and Djeddi, 2018; Islam et al., 2025; EFSA Panel on Food Additives and Flavourings, 2022). Determining the chemical profile of EOs is crucial for understanding how their main components relate to their biological effects. This is also important for ensuring that biological data can be reliably reproduced, as the composition of EOs can vary depending on the botanical origin, cultivation conditions, harvesting period, raw material processing, and variations between commercial batches. For this reason, the physicochemical characterisation and GC-MS analysis of the batch used are essential steps for experimental standardisation and for supporting the reproducibility of biological results. In the case of *Cinnamomum* sp., the specialised literature describes the existence of several chemotypes and considerable variability in volatile composition, including for commercial samples of *C. verum*, where the proportions of eugenol (E)-cinnamaldehyde, benzyl benzoate, or cinnamyl acetate can vary significantly between samples (Xavier et al., 2022). The *C. verum* EO has a high content of phenylpropanoids (91.37%), being the specific compounds according to the specialized literature (Farias et al., 2020; Xavier et al., 2022; Al-Zereini et al., 2022; Subki et al., 2013). The major compound was (E)-cinnamaldehyde (66.50%), responsible for antibacterial, antioxidant, and antifungal activity (Liu et al., 2026; de Almeida et al., 2020; Anzam and Madhavan, 2022; Molania et al., 2025). The second major compound was eugenol (19.14%), known for its antioxidant, anti-inflammatory, and antimicrobial synergy effects (Sanla-Ead et al., 2012; Marchese et al., 2017; Çali and Çelik, 2024; Barbarossa et al., 2022). Besides the main compounds, other antifungal compounds identified in the *C. verum* EO were cinnamyl acetate (3.82%), eugenol acetate (1.23%) (E)-cinnamic acid (0.25%), safrole (0.09%), and (E)-cinnamyl alcohol (0.02%) (Vizcaíno-Páez et al., 2016; Musthafa et al., 2016; Zhang et al., 2019; Li et al., 2023; He et al., 2023).

The analysis identified the presence of monoterpenes (3.67%), comprising 1.24% hydrocarbon monoterpenes and 2.43% oxygenated monoterpenes, with the main compounds being α-terpineol (0.57%), linalool (1.10%), limonene (0.38%), and p-cymene (0.35%). These compounds contribute to the aroma and may modulate biological activity (Chigo-Hernandez and Tomasino, 2023; Pyo and Jung, 2024). The sesquiterpenes that have been identified are present in a percentage of 3.75%, containing 3.57% hydrocarbon sesquiterpenes and 0.18% oxygenated sesquiterpenes. The dominant compound in this class is β-caryophyllene (2.95%), which has been shown to have an anti-inflammatory role and potential antimicrobial effect (Gushiken et al., 2022; Dickson et al., 2023).

The *C. verum* EO composition matched previous studies (Farias et al., 2020; Xavier et al., 2022; Knauth et al., 2018; Narayanankutty et al., 2021; Barbarossa et al., 2022). GC-MS analysis revealed that cinnamaldehyde and eugenol constituted the predominant constituents, accounting for approximately 85% of the total compounds detected. Despite their relatively minor concentrations, the monoterpenes and sesquiterpenes present could still influence the EO’s overall efficacy, potentially through synergistic interactions.

The physicochemical characterisation and GC-MS analysis of the batch used in the present study were essential for experimental standardisation, for correlating biological activity with the identified compositional profile, and for supporting the reproducibility of the obtained results. The compositional profile identified in this study was in accordance with previously reported data for EO of *C. verum*, suggesting that the analysed batch is representative of this species.

Stability evaluations indicated that fluconazole and *C. verum* EO exhibited favorable chemical compatibility. Recovery rates varied between 100% and 112.22%, depending on the sample storage conditions. Statistical analysis did not reveal significant differences in recovery (*p* > 0.05), suggesting that fluconazole maintained its chemical stability and detectability in the presence of *C. verum* EO throughout the study period. These results support the chemical-analytical compatibility of the combination and indicate that it can continue to be used for experimental antifungal evaluations.

The antifungal susceptibility testing of *C. verum* EO against reference and clinical *Candida* strains revealed a broad-spectrum antifungal efficacy against all tested strains compared with the solvent similar with others (Di Vito et al., 2024). Previously, Goel et al. (2016) reported that fluconazole exhibited high antifungal activity against bloodstream *Candida* isolates, with the highest susceptibility observed in *C. parapsilosis* and *C. albicans*, followed by *C. tropicalis*, whereas *C. krusei* showed complete resistance. In contrast, cinnamon EO demonstrated antifungal efficacy against all tested *Candida* species, with susceptibility observed in the following order: *C. albicans* > *C. parapsilosis* > *C. krusei* > *C. tropicalis*. Notably, cinnamon EO retained inhibitory activity against more than half of the fluconazole-resistant *C. krusei* isolates, highlighting its potential as a complementary or alternative therapeutic agent for the management of infections caused by azole-resistant *Candida* strains.

Our findings are consistent with those reported by Tran et al. (2020) regarding the efficacy of *C. verum* against reference and clinical strains; Wijesinghe et al. (2021), El-Baz et al. (2021) and Essid et al. (2017) that reported comparable MIC values.

According to prior research, *C. auris* exhibits a wide range of virulence, which is influenced by phenotypic characteristics and the various expressions of factors such filamentation, tissue invasion, and biofilm formation (Garcia-Bustos et al., 2021). However, little is known regarding the way that antifungal therapies affect these processes. The influence of *C. verum* EO on the adherence capacity of *Candida* strains to inert substratum evaluated at sub-inhibitory concentrations indicate a significant inhibition of PICA% at both tested concentrations compared to the strain control. At both MIC/2 and MIC/4 concentrations, a reduction in adherence capacity was observed for all tested strains compared with their respective untreated control strains, with highly statistically significant differences (****, *p* < 0.0001). Furthermore, the EO demonstrated high PICA%, ranging from 12.32% to 94.09%, depending on the strain and concentration. Previously, Wijesinghe et al. (2021) reported anti-biofilm activity against *C. albicans*, *C. tropicalis*, and *C. dubliniensis* strains at relatively low concentrations. Similarly, El-Baz et al. (2021) showed that treatment with *C. verum* EO significantly reduced biofilm formation capacity in *C. albicans*. Microscopic analyses further confirmed these findings, showing reduced cell adherence capacity and disruption of biofilm architecture, along with decreased cell height and surface roughness, which impair the initial attachment phase essential for biofilm formation.

The best predictions were obtained using quadratic models, as shown by the high values of the coefficient of determination (R2), especially for *C. parapsilosis* (0.8723) and *C. tropicalis* (0.8007). These results indicate the presence of nonlinear relationships and concentration dependent interactions between fluconazole and *C. verum* EO, suggesting that antifungal responses cannot be explained as simple additive effects. Clinical strains including *C. auris* R5–R12 showed lower *R* ^2^ values and higher %CV values, aspects that may be related to the inherent biological variability and the different sensitivity of the strains to the investigated combinations. Although some responses were statistically significant, *p* < 0.05, not all models were statistically significant, an aspect that highlights the complexity of the biological systems investigated. The diagnostic analysis confirmed the robustness of the models, with most studentized residuals falling within the acceptable range (±3). The values of Cook’s distance and DFFITS indicated that individual observations had little influence on the models’ performance. The variability observed for some responses could be related to the biological heterogeneity of the strains and the complexity of the antifungal response mechanisms. Fluconazole showed the highest effect on MIC value for most strains, however, some strains such as *C. auris* 1370 showed reduced sensitivity to conventional antifungal treatment suggesting an additional EO contribution to improve the antifungal effect (Supplementary Figure S1). The differences between strains may be associated with different antifungal susceptibility profiles and different resistance mechanisms, especially for *C. auris*, a species with increased azole resistance (Frías-De-León et al., 2020; Jangir et al., 2023). EO may be involved in different modes of action such as rupture of the cell membrane, modification of membrane permeability and induction of oxidative stress (Nazzaro et al., 2017; Kowalczyk, 2024). The joint effect can be attributed to complementary mechanisms such as the inhibition of ergosterol synthesis by fluconazole and the disruption of the cell membrane by the bioactive compounds of EO, especially cinnamaldehyde (Shreaz et al., 2011).

The DOE/RSM methodology combined with the desirability function allowed determining an optimal formulation with concentrations of 15.00 μg/mL fluconazole and 5.00 μL/mL *C verum* EO. Values of the MAE (1.726 mm) and RMSE (1.908 mm) obtained a good agreement between experimental and predicted values. Experimental values were slightly higher than estimated for some strains, which may suggest positive interactions between fluconazole and EO. On the other hand, the lower values observed for *C. auris* and some clinical strains may indicate increased antifungal resistance or limitations related to the experimental system used. The global desirability function (0.609) indicated a good compromise between the maximization of antifungal responses and minimization of the concentrations used. The convex profile of the response surface and the relatively narrow operating region reflect the nonlinear nature of the system and presence of trade-offs among individual responses, which is a characteristic feature of multi-response optimization. The results support the utility of the DOE/RSM methodology in the evaluation and optimization of antifungal combinations and suggest that the fluconazole–*C. verum* EO association could be a promising approach against some strains of *Candida* spp. with reduced sensitivity to conventional antifungal treatments.

Additionally, the combination of *C. verum* EO and fluconazole demonstrated mostly synergistic effects against tested strains of *Candida*, both MDR and fluconazole-resistant strains, indicating that the EO may enhance or even restore fluconazole efficacy. Previously, Di Vito et al. (2024) reported that *C. zeylanicum* EO significantly enhanced the antifungal efficacy of fluconazole against *C. auris*, leading to a reduction in MIC values. Consistent with our findings obtained for clinical *Candida* isolates, Di Vito et al. (2023) reported that all ten clinical *C*. *auris* strains exhibited high-level resistance to fluconazole, with several isolates displaying a MDR phenotype. In contrast, *C*. *zeylanicum* EO demonstrated pronounced antifungal activity against all tested strains, with MIC_90_ and MFC_90_ values of 0.06% v/v, confirming both growth-inhibitory and fungicidal effects. Notably, when combined with fluconazole, *C. zeylanicum* EO exhibited a marked synergistic interaction (FICI = 0.26 ± 0.14), effectively restoring fluconazole susceptibility at clinically relevant drug concentrations (0.45 ± 0.32 mg/mL), whereas cinnamaldehyde, the major constituent of the EO, produced only an additive effect. Further mechanistic investigations revealed that this synergistic interaction was associated with a significant reduction in fungal ATPase activity and enhanced intracellular accumulation of fluconazole, suggesting that CZ-EO may potentiate azole efficacy by interfering with ATP-dependent efflux pump activity and thereby overcoming antifungal resistance mechanisms. In addition, several studies have shown that cinnamaldehyde, the main bioactive compound in cinnamon EO, exerts antifungal activity by disrupting fungal cell membrane integrity and interfering with ergosterol biosynthesis, while also increasing membrane permeability, thereby facilitating the activity of conventional antifungal agents (Essid et al., 2017). Furthermore, Essid et al. (2017) demonstrated a clear synergistic interaction between *C. verum* EO and fluconazole, with a FICI value of 0.37 against *Candida* spp. This value falls within the same range as our obtained results (0.03 - 0.47), confirming that your observed synergy, especially very low FICI values such as 0.03 - 0.08 is even stronger than previously reported averages. Moreover, the study showed that the combination significantly reduced ergosterol content and virulence factors (secreted aspartic protease activity), explaining mechanistically why such low FICI values (high synergy) are obtained. Additionally, D’agostino et al. (2019) confirm that cinnamaldehyde synergizes with fluconazole, with reported FICI values around 0.312 – 0.37 and significant MIC reductions (up to 8-fold). These findings align closely with our obtained results, where most strains showed FICI < 0.25, indicating even stronger potentiation of fluconazole activity.

Cinnamon EOs have antibacterial activity, but their use may be limited due to their cytotoxic and hemolytic effects on host cells (Belete et al., 2025; Silva et al., 2008; Taib et al., 2009). The haemolytic effect in this study was concentration-dependent and the haemolysis values decreased gradually with decreasing concentration in all the analysed samples. At 50 and 25 μg/mL the haemolysis values were less than 1% for all the samples, showing good hemocompatibility under these conditions.

The bioactives of EO such as terpenes, phenols and aldehydes, may alter the permeability and integrity of the erythrocyte membrane (Mendanha and Alonso, 2015; Połeć et al., 2022; Tkaczenko et al., 2025; Rezende et al., 2017). Furthermore, the antibacterial activity of cinnamon EO is well documented and presents different mechanisms of action against microorganisms (Zhang et al., 2016; Iseppi et al., 2024; Abdelatti et al., 2023). However, at high concentrations, non-selectivity can harm host cells (Belete et al., 2025).

The decrease in cytotoxicity with erythrocytes due to the combination with Fluconazole:EO could be due to differences in the bioavailability of fluconazole and a different interaction with the erythrocyte membrane owing to a more homogeneous distribution of the active compound. At the same time, it is possible to induce oxidative stress in erythrocytes with fluconazole alone, as evidenced by an increase of reactive oxygen species and a decrease of antioxidant defenses, which leads to destabilization of the erythrocyte membrane and erythrolysis (Sikandar et al., 2025). In this context, the addition of EO could reduce direct interaction with the membrane and local oxidative stress. Moreover, enhancing the antifungal effect through the association with EO may allow the use of lower concentrations of fluconazole, simultaneously contributing to the reduction of cytotoxicity and the increase of antifungal efficacy.

The reference strains of *C. tropicalis*, *C. albicans*, *C. parapsilosis*, and *C. auris* were chosen for the *in vivo* investigation using *G. mellonella* model to track the expression of virulence factors and the larval mortality rate both in the absence of therapy and with different therapeutic approaches as previously shown (Mariotti et al., 2022; Wijesinghe et al., 2020). This model had been selected due to its demonstrated validity in fungal infection research and its potential to reproduce the innate immune response (Anower et al., 2025).

For hemolysin secretion, all treatments we statistically significant in terms of reduction, with the combination therapy consistently showing the strongest effect and highest level of significance, confirming the synergistic inhibitory effect as previously described (Pelliccia et al., 2024). In the *G. mellonella* model, *C. verum* EO significantly modulates lipase production, a key virulence factor in *Candida*. All treatments significantly reduced enzyme production relative to the untreated control across the tested *Candida* species, although the magnitude of inhibition varied in a species-dependent manner. It is known that fluconazole and the cinnamaldehyde can reduce the lipase secretion and to modulate the expression of secreted aspartyl proteases (SAPs), key virulence factors responsible for adherence, biofilm formation, invasion and immune evasion in *Candida* (Pelliccia et al., 2024). Regarding caseinase secretion, all treatments significantly reduced enzyme production relative to the untreated control across all *Candida* species, although the extent of inhibition was species dependent. The inhibitory effect followed the trend: fluconazole ≥ combination therapy ≥ cinnamon EO, largely driven by the pronounced activity observed in *C. auris*. As previously shown caseinase secretion is strain-dependent and it is necessary to have higher concentrations of antifungal to obtain the inhibition effect (Di Vito et al., 2024). Furthermore, different clades of *C. auris* demonstrated strain dependent activity regarding protease secretion at sub-inhibitory concentrations of cinnamon EO (Ahaik et al., 2026).

The *in vivo* study, using the *G. mellonella* model, demonstrated the superior antifungal effect of the combination of *C. verum* EO and fluconazole compared to individual monotherapies, as reflected by the significant reduction in larval mortality, fungal load, and the expression of key virulence factors such as hemolysins, lipases and caseinases. These results support the inclusion of cinnamon EO as a therapeutic adjuvant in current antifungal regimens, highlighting its ability to potentiate the effect of fluconazole against *Candida* strains and providing a solid rationale for further preclinical research on synergistic natural or synthetic combinations. While fluconazole alone demonstrated only partial efficacy and the EO alone was not fungicidal, the *C. verum* EO - fluconazole combination produced the lowest mortality rates (0%), indicating the highest protective effect; treatments decreased mortality compared to controls but may not completely eradicate the fungi and may allow survival or adaptive responses. These results align with previous investigations using the *G. mellonella* model, which reported both time-dependent mortality and variability in virulence among *Candida* species (Brennan et al., 2002; Fuchs et al., 2010). The low fungicidal effect of fluconazole aligned with several other reported studies that demonstrated its reduced efficacy and predominantly the fungistatic effect (Desbois and Coote, 2011; Lockhart et al., 2017). In addition, the absence of mortality, despite inhibition of different virulence factors, corresponds with findings indicating that EOs can modulate pathogen virulence without causing the fungicidal effect (Guimarães et al., 2019). The absence of larval mortality under low concentrations of *Cinnamomum zeylanicum* EO of exposure while the combination with fluconazole revealed the synergistic effect and significantly enhances survival in infected larvae (Di Vito et al. (2023)) highlighting that cinnamon EO represent an adjuvant agent to improve antifungal efficacy against *C. auris* infection.

## 5 Conclusion

Our study demonstrates that *C. verum* EO, exhibits antifungal efficacy against both reference and clinical MDR *C. auris* strains. The EO inhibited fungal growth and adherence capacity to inert substratum supporting its anti-virulence potential and its relevance in traditional medicine. The combination treatment demonstrated synergistic activity against most of the tested strains, while also showing reduced cytotoxic effects. The FICI values and the RSM analysis suggested synergistic effects of *C. verum* EO and fluconazole against *C. auris* strains, but further studies are necessary to fully validate the pharmacological interactions involved. In the *G. mellonella* infection model, it led to lower mortality rates, decreased fungal load, and reduced production of virulence-associated enzymes, including hemolysins, lipases, and caseinases, compared to untreated controls, suggesting both antifungal and anti-virulence potential of *C. verum* EO, that need to be better understood by mechanistic studies involving ergosterol quantification, membrane integrity and permeability assays, ROS measurements, and gene-expression analyses of resistance- and virulence-related pathways. Although the *Galleria mellonella* model provided valuable preliminary *in vivo* evidence of antifungal efficacy, further validation in mammalian infection models and clinical studies are required to confirm the therapeutic potential and translational applicability of our findings.

## Data Availability

The original contributions presented in the study are included in the article/Supplementary Material, further inquiries can be directed to the corresponding author.
