# Supplementary material for: In vitro and in vivo antifungal effects of fluconazole in combination with Cinnamomum verum essential oil against Candida spp
Source: Front Pharmacol. 2026 Jul 1;17:1864146. doi: 10.3389/fphar.2026.1864146 (PMC13369618; doi:10.3389/fphar.2026.1864146)
Supplement: Supplementary file 1 [file Supplementaryfile1.docx]

Supplementary Material

**Supplementary Table S1**. Evaluation of the concentration and recovery percentage of fluconazole treated with cinnamon EO based on various storage conditions.

| **Fluconazole mass (mg)** | **Theoretical concentration (mg/mL)** | **Peak area** | **Experimental concentration (mg/mL)** | **Recovery (%)** |
| --- | --- | --- | --- | --- |
| Dark, 4 ℃ | | | | |
| 0.30 | 0.33 | 441.40 | 0.32±0.00816 | 106.66±3.33 |
| Dark, 25 ℃ | | | | |
| 0.30 | 0.31 | 439.40 | 0.33±0.0047 | 112.22±1,92 |
| Light 25 ℃ | | | | |
| 0.30 | 0.33 | 447.40 | 0.32±0.0169 | 108.88±6,93 |
| Dark, 37 ℃ | | | | |
| 0.30 | 0.32 | 381.00 | 0.30±0.0081 | 100±3.33 |
| T0 - time of preparation | | | | |
| 0.30 | 0.31 | 422.70 | 0.32±0.0141 | 106.66±5.77 |

**Supplementary Table S2**. The average of MIC values obtained for *C. verum* EO and DMSO against *Candida* spp. strains.

| **Strains** | ***Cinnamonum verum* EO** | | **DMSO** | |
| --- | --- | --- | --- | --- |
|  | **MIC µL/mL** | **SD** | **MIC µL/mL** | **SD** |
| *C. albicans ATCC 10231* | 0,95 | 0,12 | 5 | 0 |
| *C. auris DSM 21092* | 0,72 | 0,1 | 2,5 | 0,47 |
| *C. parapsilosis ATCC 22019* | 0,88 | 0,08 | 3,75 | 1,25 |
| *C. tropicalis DSM 7524* | 0,62 | 0,11 | 7,5 | 2,5 |
| *6816* | 0,14 | 0,14 | 5 | 0 |
| *6328* | 2,88 | 0,2 | 2,5 | 0,5 |
| *4574* | 2,78 | 0,23 | 10 | 0 |
| *3396* | 0,19 | 0,19 | 1,25 | 0,5 |
| *2851* | 0,74 | 0,09 | 0,63 | 0,13 |
| *18519* | 2,85 | 0,25 | 1,25 | 0,25 |
| *1370* | 0,79 | 0,13 | 10 | 0 |
| *9069* | 2,94 | 0,27 | 2,5 | 0,3 |

**Supplementary Table S3**. Microbial adherence percentage (MAC%) values for *C. verum* EO compared to the control *Candida* strains

| **Strain** | **MIC/2** | | **MIC/4** | | **Control strain** | |
| --- | --- | --- | --- | --- | --- | --- |
|  | **Microbial adherence %** | **SD** | **Microbial adherence %** | **SD** | **Microbial adherence %** | SD |
| *C. albicans ATCC 10231* | 33,79726 | 2 | 27,06343 | 5 | 100 | 5 |
| *C. auris DSM 21092* | 17,4932 | 1 | 25,89462 | 6 | 100 | 2 |
| *C. parapsilosis ATCC 22019* | 16,9969 | 2 | 12,595 | 5 | 100 | 0,5 |
| *C. tropicalis DSM 7524* | 27,79341 | 2 | 21,63141 | 4 | 100 | 7,963829 |
| *18519* | 11,48574 | 2 | 6,938719 | 5 | 100 | 9,32 |
| *2851* | 12,9952 | 1,5 | 16,4407 | 4 | 100 | 8,25 |
| *9069* | 21,1392 | 1,5 | 15,74624 | 3 | 100 | 5,89 |
| *6328* | 40,61414 | 5 | 29,05385 | 8 | 100 | 2,34 |
| *1370* | 12,41529 | 1,5 | 7,444092 | 7 | 100 | 4,34 |
| *3396* | 9,63568 | 1,5 | 5,90872 | 4 | 100 | 6,34 |
| *6816* | 81,8397 | 1,5 | 87,67912 | 6 | 100 | 7,79 |
| *4574* | 12,75417 | 1 | 7,381597 | 3 | 100 | 5,34 |

**Table S4.** Model diagnostics for the interaction effects of fluconazole and EOs on inhibition zone diameters against *C. auris* DSM 21092

| **Run Order** | **Actual Value** | **Predicted Value** | **Residual** | **Leverage** | **Internally Studentized Residuals** | **Externally Studentized Residuals** | **Cook's Distance** | **Influence on Fitted Value DFFITS** | **Standard Order** |
| --- | --- | --- | --- | --- | --- | --- | --- | --- | --- |
| 1 | 19,00 | 23,53 | -4,53 | 0,186 | -0,663 | -0,648 | 0,033 | -0,310 | 16 |
| 2 | 31,33 | 19,82 | 11,51 | 0,069 | 1,574 | 1,681 | 0,061 | 0,458 | 8 |
| 3 | 30,66 | 18,80 | 11,86 | 0,087 | 1,638 | 1,766 | 0,085 | 0,546 | 11 |
| 4 | 15,00 | 23,59 | -8,59 | 0,186 | -1,256 | -1,287 | 0,120 | -0,615 | 15 |
| 5 | 21,00 | 16,94 | 4,06 | 0,291 | 0,636 | 0,621 | 0,055 | 0,398 | 14 |
| 6 | 41,66 | 33,36 | 8,30 | 0,260 | 1,273 | 1,307 | 0,190 | 0,775 | 12 |
| 7 | 15,00 | 12,16 | 2,84 | 0,197 | 0,418 | 0,404 | 0,014 | 0,200 | 10 |
| 8 | 21,00 | 14,44 | 6,56 | 0,185 | 0,958 | 0,955 | 0,070 | 0,456 | 3 |
| 9 | 10,33 | 11,81 | -1,48 | 0,124 | -0,209 | -0,201 | 0,002 | -0,075 | 5 |
| 10 | 21,00 | 27,99 | -6,99 | 0,200 | -1,031 | -1,034 | 0,089 | -0,518 | 9 |
| 11 | 0,0000 | 6,57 | -6,57 | 0,252 | -1,002 | -1,002 | 0,112 | -0,581 | 1 |
| 12 | 0,0000 | 6,57 | -6,57 | 0,252 | -1,002 | -1,002 | 0,112 | -0,581 | 2 |
| 13 | 20,66 | 19,87 | 0,7878 | 0,069 | 0,108 | 0,104 | 0,000 | 0,028 | 6 |
| 14 | 24,66 | 33,36 | -8,70 | 0,260 | -1,334 | -1,380 | 0,209 | -0,818 | 13 |
| 15 | 15,00 | 19,87 | -4,87 | 0,069 | -0,666 | -0,651 | 0,011 | -0,177 | 7 |
| 16 | 25,00 | 22,61 | 2,39 | 0,313 | 0,380 | 0,367 | 0,022 | 0,248 | 4 |

**Table S5.** Model diagnostics for the interaction effects of fluconazole and EOs on inhibition zone diameters against *C. albicans* 10231

| **Run Order** | **Actual Value** | **Predicted Value** | **Residual** | **Leverage** | **Internally Studentized Residuals** | **Externally Studentized Residuals** | **Cook's Distance** | **Influence on Fitted Value DFFITS** | **Standard Order** |
| --- | --- | --- | --- | --- | --- | --- | --- | --- | --- |
| 1 | 30,33 | 31,29 | -0,9574 | 0,359 | -0,115 | -0,109 | 0,001 | -0,082 | 16 |
| 2 | 21,00 | 29,12 | -8,12 | 0,216 | -0,881 | -0,871 | 0,036 | -0,457 | 8 |
| 3 | 32,66 | 31,81 | 0,8542 | 0,181 | 0,091 | 0,086 | 0,000 | 0,041 | 11 |
| 4 | 38,33 | 31,33 | 7,00 | 0,360 | 0,842 | 0,828 | 0,066 | 0,621 | 15 |
| 5 | 21,33 | 18,98 | 2,35 | 0,570 | 0,344 | 0,328 | 0,026 | 0,378 | 14 |
| 6 | 0,0000 | 19,23 | -19,23 | 0,439 | -2,468 | -3,745 | 0,794 | -3,311⁽¹⁾ | 12 |
| 7 | 5,00 | 16,90 | -11,90 | 0,503 | -1,623 | -1,794 | 0,444 | -1,804 | 10 |
| 8 | 0,0000 | 9,79 | -9,79 | 0,351 | -1,168 | -1,193 | 0,123 | -0,876 | 3 |
| 9 | 25,66 | 18,31 | 7,35 | 0,218 | 0,799 | 0,784 | 0,030 | 0,414 | 5 |
| 10 | 30,00 | 17,57 | 12,43 | 0,336 | 1,467 | 1,571 | 0,182 | 1,118 | 9 |
| 11 | 0,0000 | -3,22 | 3,22 | 0,427 | 0,409 | 0,391 | 0,021 | 0,338 | 1 |
| 12 | 0,0000 | -3,22 | 3,22 | 0,427 | 0,409 | 0,391 | 0,021 | 0,338 | 2 |
| 13 | 31,33 | 29,23 | 2,10 | 0,216 | 0,228 | 0,217 | 0,002 | 0,114 | 6 |
| 14 | 30,00 | 19,23 | 10,77 | 0,439 | 1,382 | 1,458 | 0,249 | 1,289 | 13 |
| 15 | 30,66 | 29,23 | 1,43 | 0,216 | 0,155 | 0,147 | 0,001 | 0,077 | 7 |
| 16 | 0,0000 | 0,7212 | -0,7212 | 0,743 | -0,137 | -0,130 | 0,009 | -0,221 | 4 |

⁽¹⁾ Exceeds limits.

**Table S6**. Model diagnostics for the interaction effects of fluconazole and EOs on inhibition zone diameters against *C. parapsilosis* ATCC 22019

| **Run Order** | **Actual Value** | **Predicted Value** | **Residual** | **Leverage** | **Internally Studentized Residuals** | **Externally Studentized Residuals** | **Cook's Distance** | **Influence on Fitted Value DFFITS** | **Standard Order** |
| --- | --- | --- | --- | --- | --- | --- | --- | --- | --- |
| 1 | 41,00 | 35,20 | 5,80 | 0,359 | 1,341 | 1,405 | 0,168 | 1,051 | 16 |
| 2 | 39,66 | 44,35 | -4,69 | 0,216 | -0,981 | -0,979 | 0,044 | -0,513 | 8 |
| 3 | 38,33 | 35,30 | 3,03 | 0,181 | 0,621 | 0,601 | 0,014 | 0,283 | 11 |
| 4 | 35,00 | 35,37 | -0,3692 | 0,360 | -0,085 | -0,081 | 0,001 | -0,061 | 15 |
| 5 | 7,00 | 9,20 | -2,20 | 0,570 | -0,621 | -0,600 | 0,085 | -0,691 | 14 |
| 6 | 45,00 | 45,70 | -0,7010 | 0,439 | -0,173 | -0,165 | 0,004 | -0,146 | 12 |
| 7 | 8,33 | 11,63 | -3,30 | 0,503 | -0,866 | -0,854 | 0,126 | -0,859 | 10 |
| 8 | 40,66 | 44,82 | -4,16 | 0,351 | -0,957 | -0,952 | 0,082 | -0,700 | 3 |
| 9 | 31,33 | 28,70 | 2,63 | 0,218 | 0,552 | 0,532 | 0,014 | 0,281 | 5 |
| 10 | 50,00 | 42,79 | 7,21 | 0,336 | 1,639 | 1,818 | 0,226 | 1,293 | 9 |
| 11 | 31,33 | 29,15 | 2,18 | 0,427 | 0,534 | 0,514 | 0,035 | 0,444 | 1 |
| 12 | 30,00 | 29,15 | 0,8540 | 0,427 | 0,209 | 0,199 | 0,005 | 0,172 | 2 |
| 13 | 50,66 | 44,34 | 6,32 | 0,216 | 1,321 | 1,380 | 0,080 | 0,725 | 6 |
| 14 | 41,33 | 45,70 | -4,37 | 0,439 | -1,081 | -1,091 | 0,152 | -0,964 | 13 |
| 15 | 36,00 | 44,34 | -8,34 | 0,216 | -1,746 | -1,986 | 0,140 | -1,043 | 7 |
| 16 | 40,00 | 39,90 | 0,1016 | 0,743 | 0,037 | 0,035 | 0,001 | 0,060 | 4 |

**Table S7**. Model diagnostics for the interaction effects of fluconazole and EOs on inhibition zone diameters against *C. tropicalis* DSM 7524

| **Run Order** | **Actual Value** | **Predicted Value** | **Residual** | **Leverage** | **Internally Studentized Residuals** | **Externally Studentized Residuals** | **Cook's Distance** | **Influence on Fitted Value DFFITS** | **Standard Order** |
| --- | --- | --- | --- | --- | --- | --- | --- | --- | --- |
| 1 | 35,33 | 31,47 | 3,86 | 0,359 | 0,611 | 0,591 | 0,035 | 0,442 | 16 |
| 2 | 38,00 | 28,28 | 9,72 | 0,216 | 1,391 | 1,469 | 0,089 | 0,771 | 8 |
| 3 | 28,00 | 28,91 | -0,9100 | 0,181 | -0,127 | -0,121 | 0,001 | -0,057 | 11 |
| 4 | 30,33 | 31,57 | -1,24 | 0,360 | -0,196 | -0,186 | 0,004 | -0,140 | 15 |
| 5 | 10,00 | 8,87 | 1,13 | 0,570 | 0,219 | 0,208 | 0,011 | 0,240 | 14 |
| 6 | 3,33 | 16,39 | -13,06 | 0,439 | -2,209 | -2,928 | 0,636 | -2,589⁽¹⁾ | 12 |
| 7 | 0,0000 | 4,73 | -4,73 | 0,503 | -0,850 | -0,837 | 0,122 | -0,842 | 10 |
| 8 | 0,0000 | 12,93 | -12,93 | 0,351 | -2,032 | -2,516 | 0,371 | -1,848⁽¹⁾ | 3 |
| 9 | 10,00 | 13,34 | -3,34 | 0,218 | -0,479 | -0,460 | 0,011 | -0,243 | 5 |
| 10 | 12,33 | 11,25 | 1,08 | 0,336 | 0,167 | 0,159 | 0,002 | 0,113 | 9 |
| 11 | 0,0000 | -4,04 | 4,04 | 0,427 | 0,676 | 0,656 | 0,057 | 0,566 | 1 |
| 12 | 0,0000 | -4,04 | 4,04 | 0,427 | 0,676 | 0,656 | 0,057 | 0,566 | 2 |
| 13 | 30,00 | 28,38 | 1,62 | 0,216 | 0,232 | 0,221 | 0,002 | 0,116 | 6 |
| 14 | 25,66 | 16,39 | 9,27 | 0,439 | 1,567 | 1,712 | 0,320 | 1,513 | 13 |
| 15 | 26,33 | 28,38 | -2,05 | 0,216 | -0,293 | -0,279 | 0,004 | -0,147 | 7 |
| 16 | 0,0000 | -3,50 | 3,50 | 0,743 | 0,874 | 0,863 | 0,368 | 1,467 | 4 |

⁽¹⁾ Exceeds limits.

**Table S8**. Model diagnostics for the interaction effects of fluconazole and EOs on inhibition zone diameters against *C. auris* 2851

| **Run Order** | **Actual Value** | **Predicted Value** | **Residual** | **Leverage** | **Internally Studentized Residuals** | **Externally Studentized Residuals** | **Cook's Distance** | **Influence on Fitted Value DFFITS** | **Standard Order** |
| --- | --- | --- | --- | --- | --- | --- | --- | --- | --- |
| 1 | 32,33 | 20,80 | 11,53 | 0,359 | 1,699 | 1,911 | 0,269 | 1,430 | 16 |
| 2 | 8,00 | 22,46 | -14,46 | 0,216 | -1,927 | -2,305 | 0,170 | -1,209 | 8 |
| 3 | 20,66 | 23,96 | -3,30 | 0,181 | -0,431 | -0,413 | 0,007 | -0,194 | 11 |
| 4 | 17,33 | 20,83 | -3,50 | 0,360 | -0,516 | -0,496 | 0,025 | -0,372 | 15 |
| 5 | 7,66 | 11,59 | -3,93 | 0,570 | -0,706 | -0,687 | 0,110 | -0,791 | 14 |
| 6 | 1,0000 | 10,71 | -9,71 | 0,439 | -1,530 | -1,658 | 0,305 | -1,466 | 12 |
| 7 | 13,33 | 12,74 | 0,5945 | 0,503 | 0,099 | 0,094 | 0,002 | 0,095 | 10 |
| 8 | 0,0000 | 7,21 | -7,21 | 0,351 | -1,055 | -1,062 | 0,100 | -0,780 | 3 |
| 9 | 13,33 | 14,79 | -1,46 | 0,218 | -0,195 | -0,185 | 0,002 | -0,098 | 5 |
| 10 | 17,00 | 11,88 | 5,12 | 0,336 | 0,742 | 0,724 | 0,046 | 0,515 | 9 |
| 11 | 0,0000 | -2,38 | 2,38 | 0,427 | 0,371 | 0,355 | 0,017 | 0,306 | 1 |
| 12 | 0,0000 | -2,38 | 2,38 | 0,427 | 0,371 | 0,355 | 0,017 | 0,306 | 2 |
| 13 | 32,00 | 22,54 | 9,46 | 0,216 | 1,261 | 1,304 | 0,073 | 0,685 | 6 |
| 14 | 15,00 | 10,71 | 4,29 | 0,439 | 0,675 | 0,656 | 0,059 | 0,580 | 13 |
| 15 | 29,00 | 22,54 | 6,46 | 0,216 | 0,861 | 0,849 | 0,034 | 0,446 | 7 |
| 16 | 0,0000 | -1,35 | 1,35 | 0,743 | 0,313 | 0,299 | 0,047 | 0,508 | 4 |

**Table S9.** Model diagnostics for the interaction effects of fluconazole and EOs on inhibition zone diameters against *C. auris* 3896

| **Run Order** | **Actual Value** | **Predicted Value** | **Residual** | **Leverage** | **Internally Studentized Residuals** | **Externally Studentized Residuals** | **Cook's Distance** | **Influence on Fitted Value DFFITS** | **Standard Order** |
| --- | --- | --- | --- | --- | --- | --- | --- | --- | --- |
| 1 | 30,66 | 24,48 | 6,18 | 0,359 | 0,766 | 0,749 | 0,055 | 0,560 | 16 |
| 2 | 8,33 | 23,47 | -15,14 | 0,216 | -1,696 | -1,906 | 0,132 | -1,000 | 8 |
| 3 | 27,33 | 25,60 | 1,73 | 0,181 | 0,190 | 0,180 | 0,001 | 0,085 | 11 |
| 4 | 24,00 | 24,51 | -0,5130 | 0,360 | -0,064 | -0,060 | 0,000 | -0,045 | 15 |
| 5 | 16,00 | 15,07 | 0,9314 | 0,570 | 0,141 | 0,134 | 0,004 | 0,154 | 14 |
| 6 | 0,0000 | 17,01 | -17,01 | 0,439 | -2,251 | -3,041 | 0,660 | -2,689⁽¹⁾ | 12 |
| 7 | 5,00 | 14,51 | -9,51 | 0,503 | -1,338 | -1,400 | 0,302 | -1,408 | 10 |
| 8 | 0,0000 | 7,14 | -7,14 | 0,351 | -0,879 | -0,868 | 0,069 | -0,638 | 3 |
| 9 | 22,66 | 15,26 | 7,40 | 0,218 | 0,830 | 0,816 | 0,032 | 0,431 | 5 |
| 10 | 26,00 | 15,37 | 10,63 | 0,336 | 1,293 | 1,344 | 0,141 | 0,956 | 9 |
| 11 | 0,0000 | -2,23 | 2,23 | 0,427 | 0,292 | 0,279 | 0,011 | 0,241 | 1 |
| 12 | 0,0000 | -2,23 | 2,23 | 0,427 | 0,292 | 0,279 | 0,011 | 0,241 | 2 |
| 13 | 28,33 | 23,57 | 4,76 | 0,216 | 0,533 | 0,513 | 0,013 | 0,269 | 6 |
| 14 | 27,00 | 17,01 | 9,99 | 0,439 | 1,322 | 1,381 | 0,228 | 1,221 | 13 |
| 15 | 27,33 | 23,57 | 3,76 | 0,216 | 0,421 | 0,403 | 0,008 | 0,212 | 7 |
| 16 | 0,0000 | 0,5288 | -0,5288 | 0,743 | -0,103 | -0,098 | 0,005 | -0,167 | 4 |

⁽¹⁾ Exceeds limits.

**Table S10**. Model diagnostics for the interaction effects of fluconazole and EOs on inhibition zone diameters against *C. auris* 1370

| **Run Order** | **Actual Value** | **Predicted Value** | **Residual** | **Leverage** | **Internally Studentized Residuals** | **Externally Studentized Residuals** | **Cook's Distance** | **Influence on Fitted Value DFFITS** | **Standard Order** |
| --- | --- | --- | --- | --- | --- | --- | --- | --- | --- |
| 1 | 17,33 | 21,49 | -4,16 | 0,186 | -0,362 | -0,350 | 0,010 | -0,167 | 16 |
| 2 | 8,66 | 12,28 | -3,62 | 0,069 | -0,295 | -0,284 | 0,002 | -0,077 | 8 |
| 3 | 15,00 | 16,82 | -1,82 | 0,087 | -0,150 | -0,144 | 0,001 | -0,045 | 11 |
| 4 | 25,00 | 21,48 | 3,52 | 0,186 | 0,307 | 0,296 | 0,007 | 0,141 | 15 |
| 5 | 20,00 | 22,75 | -2,75 | 0,291 | -0,257 | -0,247 | 0,009 | -0,158 | 14 |
| 6 | 0,0000 | 17,06 | -17,06 | 0,260 | -1,558 | -1,660 | 0,285 | -0,984 | 12 |
| 7 | 12,33 | 17,72 | -5,39 | 0,197 | -0,472 | -0,458 | 0,018 | -0,226 | 10 |
| 8 | 0,0000 | 6,24 | -6,24 | 0,185 | -0,543 | -0,528 | 0,022 | -0,252 | 3 |
| 9 | 5,00 | 12,69 | -7,69 | 0,124 | -0,645 | -0,630 | 0,020 | -0,237 | 5 |
| 10 | 0,0000 | 10,87 | -10,87 | 0,200 | -0,955 | -0,951 | 0,076 | -0,476 | 9 |
| 11 | 15,33 | 7,75 | 7,58 | 0,252 | 0,689 | 0,674 | 0,053 | 0,391 | 1 |
| 12 | 0,0000 | 7,75 | -7,75 | 0,252 | -0,704 | -0,689 | 0,055 | -0,400 | 2 |
| 13 | 42,00 | 12,34 | 29,66 | 0,069 | 2,415 | 3,125 | 0,144 | 0,850 | 6 |
| 14 | 30,66 | 17,06 | 13,60 | 0,260 | 1,242 | 1,271 | 0,181 | 0,754 | 13 |
| 15 | 30,00 | 12,34 | 17,66 | 0,069 | 1,438 | 1,507 | 0,051 | 0,410 | 7 |
| 16 | 0,0000 | 4,68 | -4,68 | 0,313 | -0,443 | -0,429 | 0,030 | -0,290 | 4 |

**Table S11.** Model diagnostics for the interaction effects of fluconazole and EOs on inhibition zone diameters against *C. auris* 9069

| **Run Order** | **Actual Value** | **Predicted Value** | **Residual** | **Leverage** | **Internally Studentized Residuals** | **Externally Studentized Residuals** | **Cook's Distance** | **Influence on Fitted Value DFFITS** | **Standard Order** |
| --- | --- | --- | --- | --- | --- | --- | --- | --- | --- |
| 1 | 21,00 | 17,71 | 3,29 | 0,359 | 0,488 | 0,468 | 0,022 | 0,350 | 16 |
| 2 | 0,0000 | 15,89 | -15,89 | 0,216 | -2,128 | -2,729 | 0,208 | -1,431 | 8 |
| 3 | 21,33 | 18,97 | 2,36 | 0,181 | 0,309 | 0,295 | 0,004 | 0,139 | 11 |
| 4 | 15,00 | 17,70 | -2,70 | 0,360 | -0,401 | -0,384 | 0,015 | -0,288 | 15 |
| 5 | 15,33 | 14,22 | 1,11 | 0,570 | 0,200 | 0,191 | 0,009 | 0,219 | 14 |
| 6 | 0,0000 | 7,72 | -7,72 | 0,439 | -1,221 | -1,256 | 0,194 | -1,111 | 12 |
| 7 | 7,00 | 13,85 | -6,85 | 0,503 | -1,151 | -1,173 | 0,223 | -1,179 | 10 |
| 8 | 0,0000 | 4,28 | -4,28 | 0,351 | -0,630 | -0,610 | 0,036 | -0,448 | 3 |
| 9 | 22,00 | 12,68 | 9,32 | 0,218 | 1,250 | 1,291 | 0,073 | 0,682 | 5 |
| 10 | 7,33 | 7,45 | -0,1204 | 0,336 | -0,018 | -0,017 | 0,000 | -0,012 | 9 |
| 11 | 0,0000 | 0,0687 | -0,0687 | 0,427 | -0,011 | -0,010 | 0,000 | -0,009 | 1 |
| 12 | 0,0000 | 0,0687 | -0,0687 | 0,427 | -0,011 | -0,010 | 0,000 | -0,009 | 2 |
| 13 | 15,00 | 15,96 | -0,9570 | 0,216 | -0,128 | -0,122 | 0,001 | -0,064 | 6 |
| 14 | 15,00 | 7,72 | 7,28 | 0,439 | 1,153 | 1,175 | 0,173 | 1,038 | 13 |
| 15 | 28,66 | 15,96 | 12,70 | 0,216 | 1,701 | 1,915 | 0,133 | 1,006 | 7 |
| 16 | 0,0000 | -2,59 | 2,59 | 0,743 | 0,605 | 0,584 | 0,176 | 0,993 | 4 |

**Table S12**. Model diagnostics for the interaction effects of fluconazole and EOs on inhibition zone diameters against *C. auris* 18519

| **Run Order** | **Actual Value** | **Predicted Value** | **Residual** | **Leverage** | **Internally Studentized Residuals** | **Externally Studentized Residuals** | **Cook's Distance** | **Influence on Fitted Value DFFITS** | **Standard Order** |
| --- | --- | --- | --- | --- | --- | --- | --- | --- | --- |
| 1 | 25,00 | 25,19 | -0,1931 | 0,359 | -0,041 | -0,039 | 0,000 | -0,029 | 16 |
| 2 | 12,00 | 18,03 | -6,03 | 0,216 | -1,161 | -1,184 | 0,062 | -0,621 | 8 |
| 3 | 25,33 | 22,32 | 3,01 | 0,181 | 0,568 | 0,548 | 0,012 | 0,258 | 11 |
| 4 | 30,33 | 25,18 | 5,15 | 0,360 | 1,097 | 1,110 | 0,113 | 0,833 | 15 |
| 5 | 20,00 | 21,27 | -1,27 | 0,570 | -0,331 | -0,316 | 0,024 | -0,363 | 14 |
| 6 | 0,0000 | 9,02 | -9,02 | 0,439 | -2,053 | -2,561 | 0,549 | -2,264⁽¹⁾ | 12 |
| 7 | 12,33 | 14,64 | -2,31 | 0,503 | -0,558 | -0,538 | 0,052 | -0,541 | 10 |
| 8 | 0,0000 | 6,13 | -6,13 | 0,351 | -1,297 | -1,349 | 0,151 | -0,991 | 3 |
| 9 | 7,00 | 11,92 | -4,92 | 0,218 | -0,950 | -0,945 | 0,042 | -0,499 | 5 |
| 10 | 17,66 | 8,58 | 9,08 | 0,336 | 1,900 | 2,256 | 0,305 | 1,605 | 9 |
| 11 | 0,0000 | -3,41 | 3,41 | 0,427 | 0,768 | 0,751 | 0,073 | 0,648 | 1 |
| 12 | 0,0000 | -3,41 | 3,41 | 0,427 | 0,768 | 0,751 | 0,073 | 0,648 | 2 |
| 13 | 20,33 | 18,11 | 2,22 | 0,216 | 0,428 | 0,410 | 0,008 | 0,215 | 6 |
| 14 | 11,33 | 9,02 | 2,31 | 0,439 | 0,526 | 0,507 | 0,036 | 0,448 | 13 |
| 15 | 20,33 | 18,11 | 2,22 | 0,216 | 0,428 | 0,410 | 0,008 | 0,215 | 7 |
| 16 | 0,0000 | 0,9351 | -0,9351 | 0,743 | -0,314 | -0,300 | 0,048 | -0,510 | 4 |

⁽¹⁾ Exceeds limits.

**Table S13**. Model diagnostics for the interaction effects of fluconazole and EOs on inhibition zone diameters against *C. auris* 6816

| **Run Order** | **Actual Value** | **Predicted Value** | **Residual** | **Leverage** | **Internally Studentized Residuals** | **Externally Studentized Residuals** | **Cook's Distance** | **Influence on Fitted Value DFFITS** | **Standard Order** |
| --- | --- | --- | --- | --- | --- | --- | --- | --- | --- |
| 1 | 12,66 | 9,51 | 3,15 | 0,186 | 0,833 | 0,823 | 0,053 | 0,394 | 16 |
| 2 | 7,66 | 3,81 | 3,85 | 0,069 | 0,953 | 0,949 | 0,023 | 0,259 | 8 |
| 3 | 7,00 | 6,49 | 0,5101 | 0,087 | 0,128 | 0,123 | 0,001 | 0,038 | 11 |
| 4 | 11,33 | 9,51 | 1,82 | 0,186 | 0,482 | 0,467 | 0,018 | 0,223 | 15 |
| 5 | 7,33 | 9,96 | -2,63 | 0,291 | -0,745 | -0,732 | 0,076 | -0,469 | 14 |
| 6 | 11,00 | 7,33 | 3,67 | 0,260 | 1,020 | 1,022 | 0,122 | 0,606 | 12 |
| 7 | 5,66 | 6,71 | -1,05 | 0,197 | -0,280 | -0,270 | 0,006 | -0,133 | 10 |
| 8 | 0,0000 | -0,0647 | 0,0647 | 0,185 | 0,017 | 0,016 | 0,000 | 0,008 | 3 |
| 9 | 0,0000 | 3,68 | -3,68 | 0,124 | -0,939 | -0,934 | 0,041 | -0,351 | 5 |
| 10 | 0,0000 | 3,36 | -3,36 | 0,200 | -0,898 | -0,891 | 0,067 | -0,446 | 9 |
| 11 | 0,0000 | 0,4624 | -0,4624 | 0,252 | -0,128 | -0,123 | 0,002 | -0,071 | 1 |
| 12 | 0,0000 | 0,4624 | -0,4624 | 0,252 | -0,128 | -0,123 | 0,002 | -0,071 | 2 |
| 13 | 13,00 | 3,85 | 9,15 | 0,069 | 2,266 | 2,799 | 0,127 | 0,761 | 6 |
| 14 | 0,0000 | 7,33 | -7,33 | 0,260 | -2,036 | -2,371 | 0,486 | -1,406⁽¹⁾ | 13 |
| 15 | 0,0000 | 3,85 | -3,85 | 0,069 | -0,954 | -0,950 | 0,022 | -0,258 | 7 |
| 16 | 0,0000 | -0,6112 | 0,6112 | 0,313 | 0,176 | 0,170 | 0,005 | 0,115 | 4 |

⁽¹⁾ Exceeds limits.

**Table S14**. Model diagnostics for the interaction effects of fluconazole and EOs on inhibition zone diameters against *C. auris* 4574

| **Run Order** | **Actual Value** | **Predicted Value** | **Residual** | **Leverage** | **Internally Studentized Residuals** | **Externally Studentized Residuals** | **Cook's Distance** | **Influence on Fitted Value DFFITS** | **Standard Order** |
| --- | --- | --- | --- | --- | --- | --- | --- | --- | --- |
| 1 | 9,33 | 9,16 | 0,1694 | 0,186 | 0,040 | 0,038 | 0,000 | 0,018 | 16 |
| 2 | 8,66 | 4,32 | 4,34 | 0,069 | 0,949 | 0,945 | 0,022 | 0,258 | 8 |
| 3 | 10,00 | 6,49 | 3,51 | 0,087 | 0,776 | 0,763 | 0,019 | 0,236 | 11 |
| 4 | 13,00 | 9,16 | 3,84 | 0,186 | 0,898 | 0,891 | 0,061 | 0,426 | 15 |
| 5 | 5,33 | 9,28 | -3,95 | 0,291 | -0,989 | -0,988 | 0,134 | -0,633 | 14 |
| 6 | 10,33 | 7,73 | 2,60 | 0,260 | 0,638 | 0,623 | 0,048 | 0,369 | 12 |
| 7 | 4,00 | 6,42 | -2,42 | 0,197 | -0,570 | -0,555 | 0,026 | -0,274 | 10 |
| 8 | 0,0000 | 0,9226 | -0,9226 | 0,185 | -0,216 | -0,208 | 0,004 | -0,099 | 3 |
| 9 | 0,0000 | 3,91 | -3,91 | 0,124 | -0,881 | -0,873 | 0,037 | -0,328 | 5 |
| 10 | 0,0000 | 4,25 | -4,25 | 0,200 | -1,004 | -1,005 | 0,084 | -0,503 | 9 |
| 11 | 0,0000 | 1,06 | -1,06 | 0,252 | -0,259 | -0,249 | 0,007 | -0,144 | 1 |
| 12 | 0,0000 | 1,06 | -1,06 | 0,252 | -0,259 | -0,249 | 0,007 | -0,144 | 2 |
| 13 | 15,33 | 4,35 | 10,98 | 0,069 | 2,401 | 3,092 | 0,142 | 0,841 | 6 |
| 14 | 0,0000 | 7,73 | -7,73 | 0,260 | -1,897 | -2,143 | 0,422 | -1,271 | 13 |
| 15 | 5,00 | 4,35 | 0,6455 | 0,069 | 0,141 | 0,136 | 0,000 | 0,037 | 7 |
| 16 | 0,0000 | 0,7801 | -0,7801 | 0,313 | -0,199 | -0,191 | 0,006 | -0,129 | 4 |

**Table S15**. Model diagnostics for the interaction effects of fluconazole and EOs on inhibition zone diameters against *C. auris* 6328

| **Run Order** | **Actual Value** | **Predicted Value** | **Residual** | **Leverage** | **Internally Studentized Residuals** | **Externally Studentized Residuals** | **Cook's Distance** | **Influence on Fitted Value DFFITS** | **Standard Order** |
| --- | --- | --- | --- | --- | --- | --- | --- | --- | --- |
| 1 | 8,00 | 11,31 | -3,31 | 0,186 | -0,526 | -0,511 | 0,021 | -0,244 | 16 |
| 2 | 10,66 | 5,34 | 5,32 | 0,069 | 0,791 | 0,779 | 0,015 | 0,212 | 8 |
| 3 | 6,66 | 8,18 | -1,52 | 0,087 | -0,228 | -0,219 | 0,002 | -0,068 | 11 |
| 4 | 11,00 | 11,30 | -0,3040 | 0,186 | -0,048 | -0,046 | 0,000 | -0,022 | 15 |
| 5 | 15,00 | 11,86 | 3,14 | 0,291 | 0,536 | 0,521 | 0,039 | 0,333 | 14 |
| 6 | 15,33 | 8,88 | 6,45 | 0,260 | 1,075 | 1,082 | 0,136 | 0,642 | 12 |
| 7 | 5,00 | 8,49 | -3,49 | 0,197 | -0,559 | -0,543 | 0,025 | -0,269 | 10 |
| 8 | 0,0000 | 1,32 | -1,32 | 0,185 | -0,210 | -0,202 | 0,003 | -0,096 | 3 |
| 9 | 4,33 | 5,30 | -0,9679 | 0,124 | -0,148 | -0,143 | 0,001 | -0,054 | 5 |
| 10 | 0,0000 | 4,76 | -4,76 | 0,200 | -0,764 | -0,751 | 0,049 | -0,376 | 9 |
| 11 | 0,0000 | 1,97 | -1,97 | 0,252 | -0,327 | -0,316 | 0,012 | -0,183 | 1 |
| 12 | 0,0000 | 1,97 | -1,97 | 0,252 | -0,327 | -0,316 | 0,012 | -0,183 | 2 |
| 13 | 25,00 | 5,38 | 19,62 | 0,069 | 2,917 | 4,768⁽¹⁾ | 0,210 | 1,297 | 6 |
| 14 | 0,0000 | 8,88 | -8,88 | 0,260 | -1,482 | -1,561 | 0,257 | -0,926 | 13 |
| 15 | 0,0000 | 5,38 | -5,38 | 0,069 | -0,800 | -0,788 | 0,016 | -0,214 | 7 |
| 16 | 0,0000 | 0,6433 | -0,6433 | 0,313 | -0,111 | -0,107 | 0,002 | -0,072 | 4 |

⁽¹⁾ Observation with |External Stud. Residuals| > 3,68

| *C. auris* DSM 21092 | 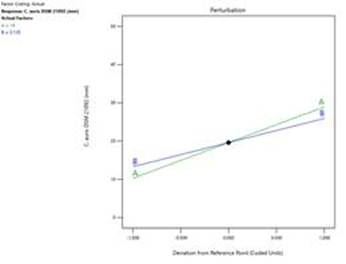 | 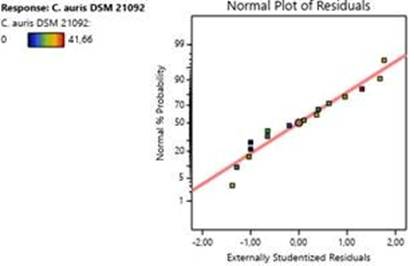 | 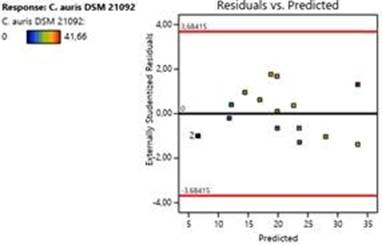 | 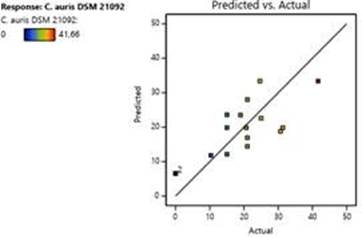 |
| --- | --- | --- | --- | --- |
| *C. albicans* ATCC 10231 | 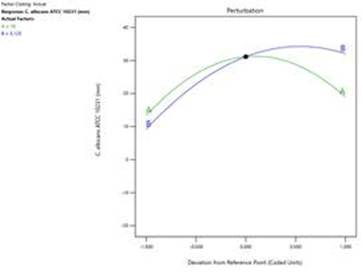 | 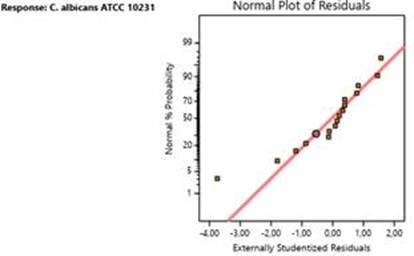 | 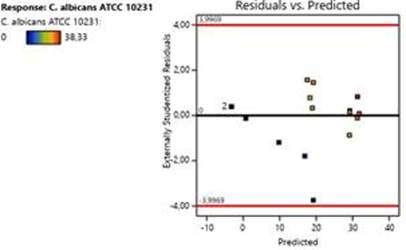 | 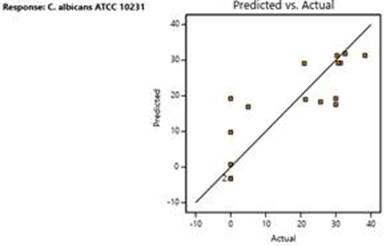 |
| *C. parapsilosis* ATCC 22019 | 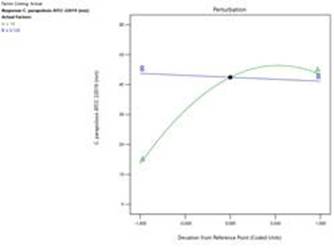 | 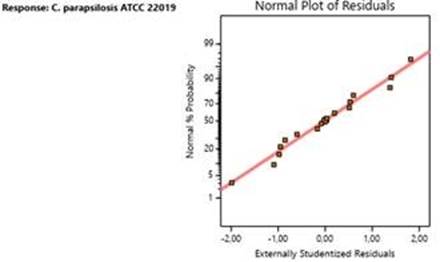 | 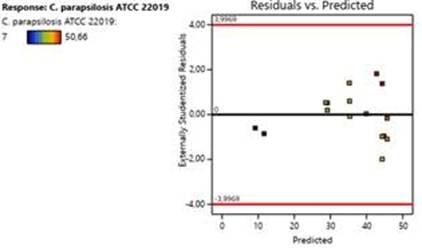 | 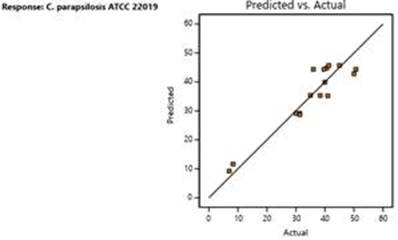 |
| *C. tropicalis* DSM 7524 | 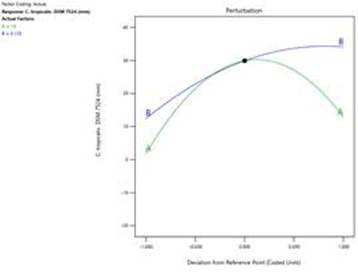 | 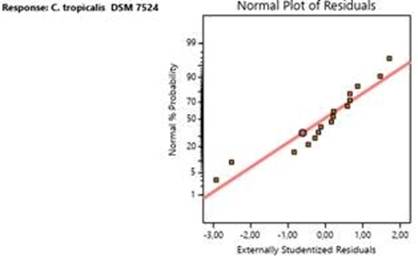 | 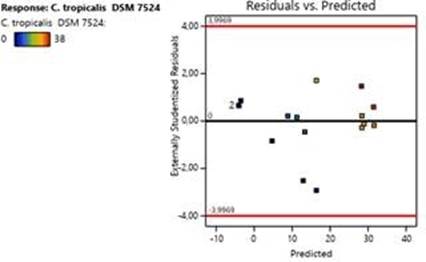 | 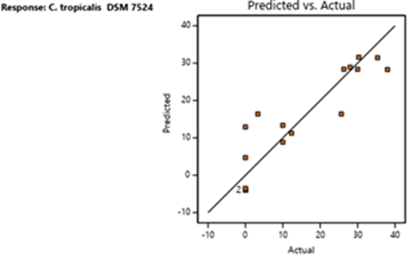 |
| 2851 | 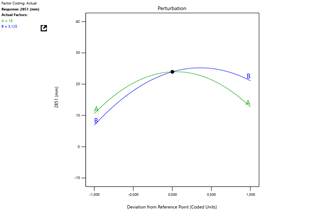 | 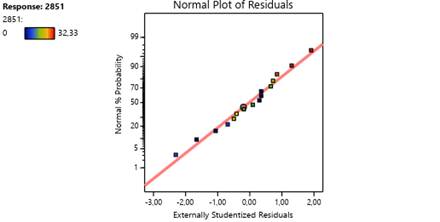 | 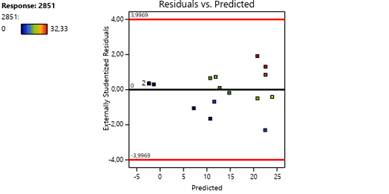 | 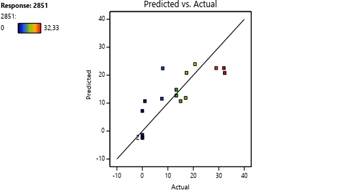 |
| 3896 | 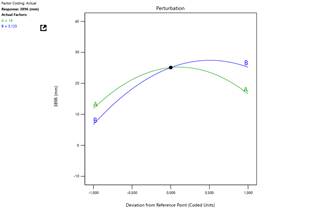 | 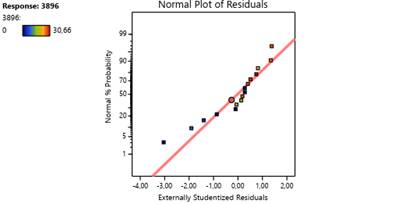 | 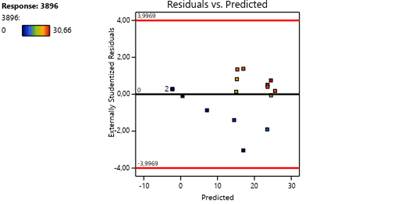 | 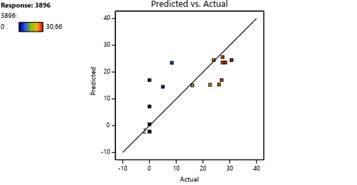 |
| 1370 | 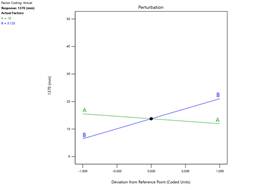 | 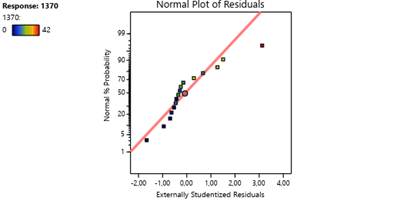 | 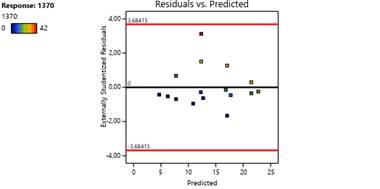 | 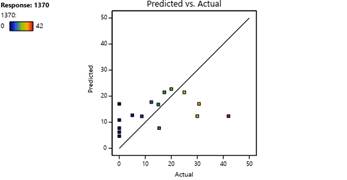 |
| 9069 | 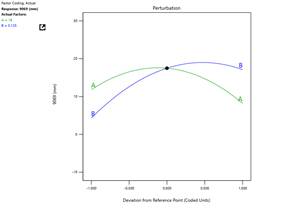 | 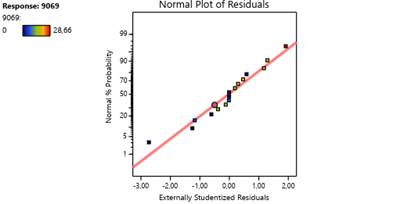 | 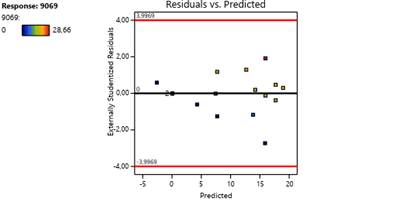 | 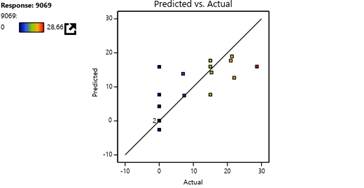 |
| 18519 | 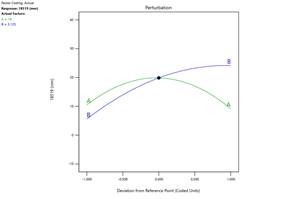 | 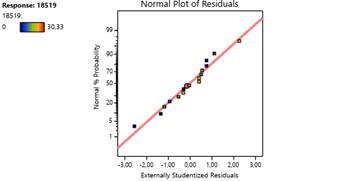 | 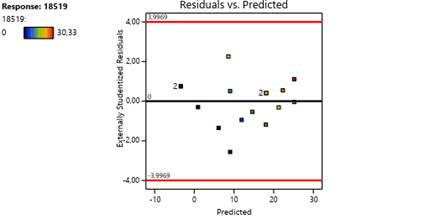 | 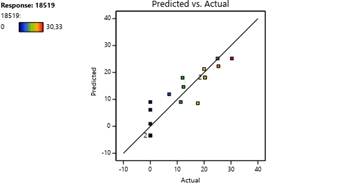 |
| 6816 | 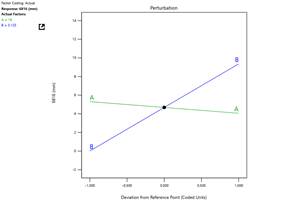 | 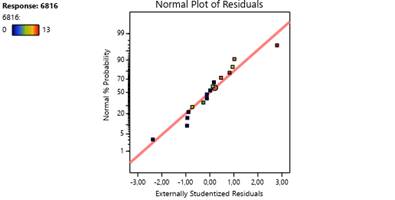 | 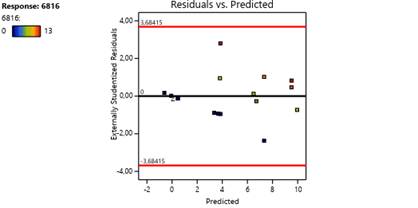 | 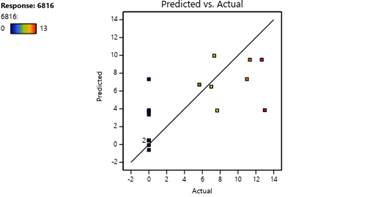 |
| 4574 | 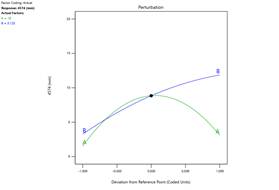 | 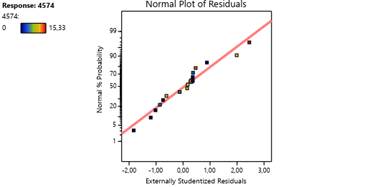 | 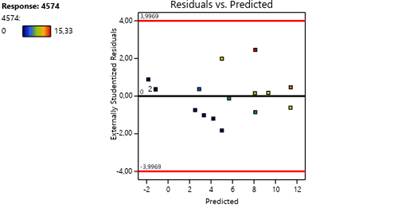 | 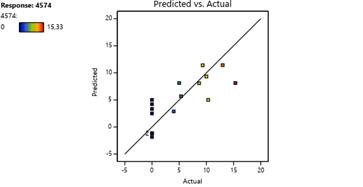 |
| 6328 | 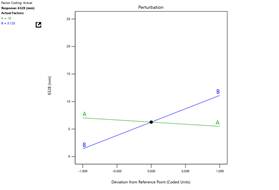 | 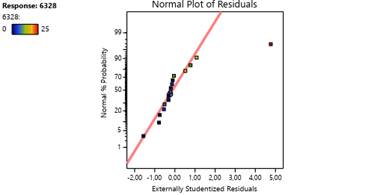 | 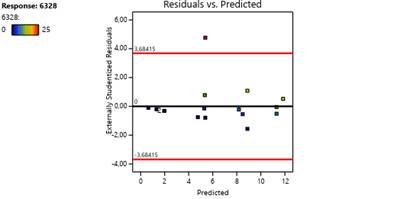 | 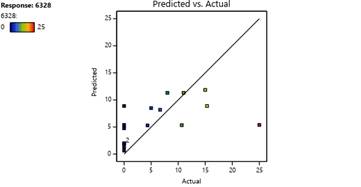 |

Figure S1. Model diagnostic plots (residuals, residuals distribution, residuals vs. predicted values, and predicted vs. experimental values) for evaluating the interaction between fluconazole and EO on the diameter of inhibition zones specific to each *Candida* spp. strain.

| 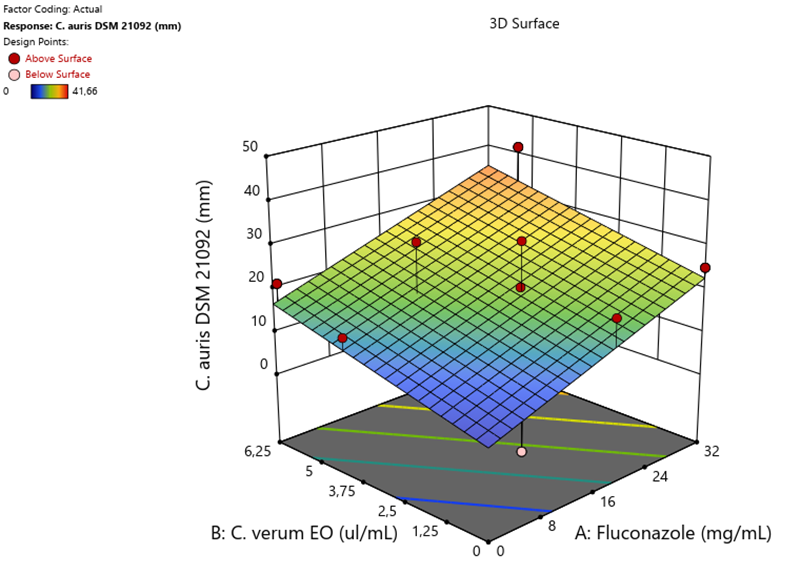 | 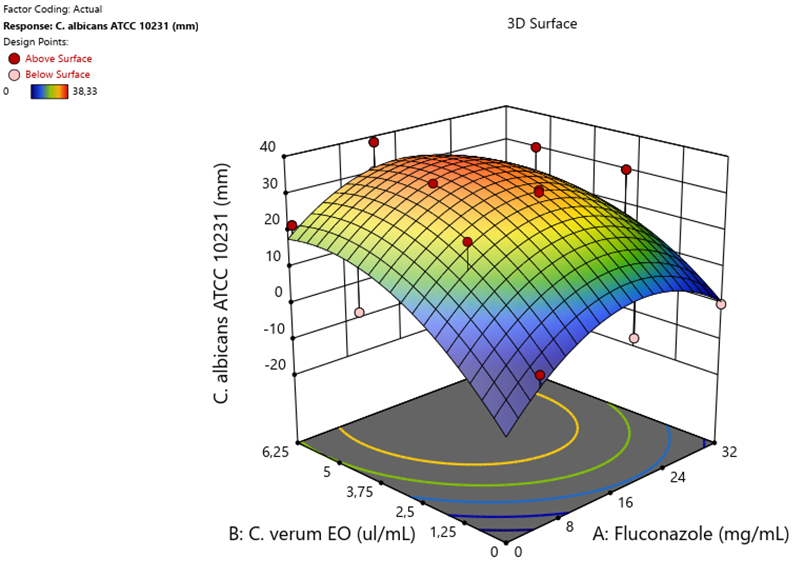 | 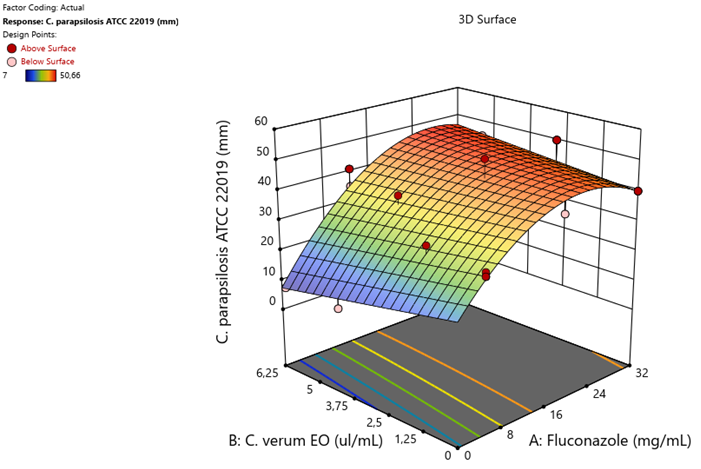 | **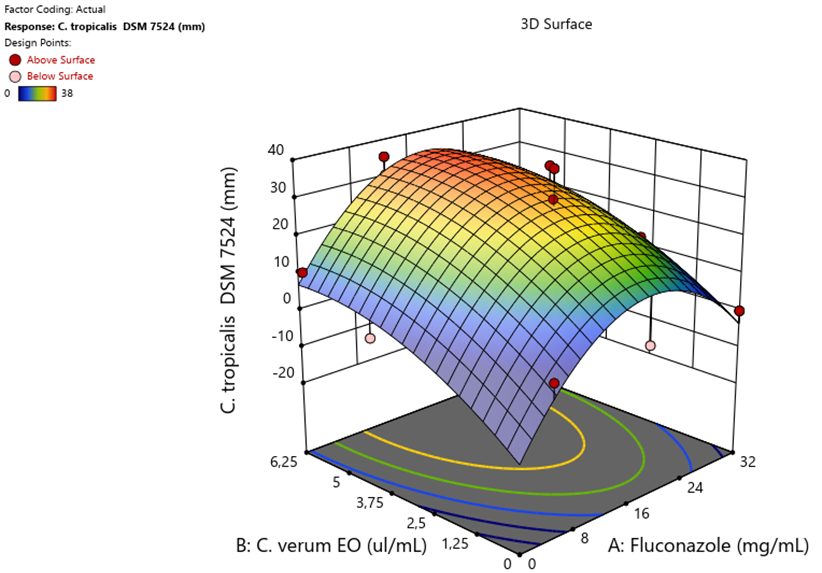** |
| --- | --- | --- | --- |
| *C. auris* DSM 21092 | *C. albicans* ATCC 1023 | *C. parapsilosis* ATCC 22019 | *C. tropicalis* DSM 7524 |
| 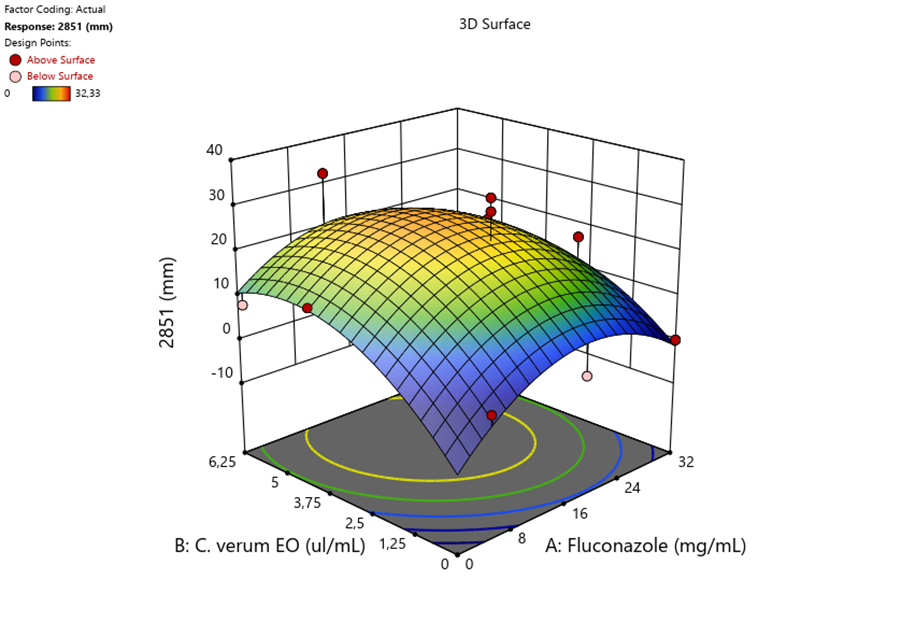 | 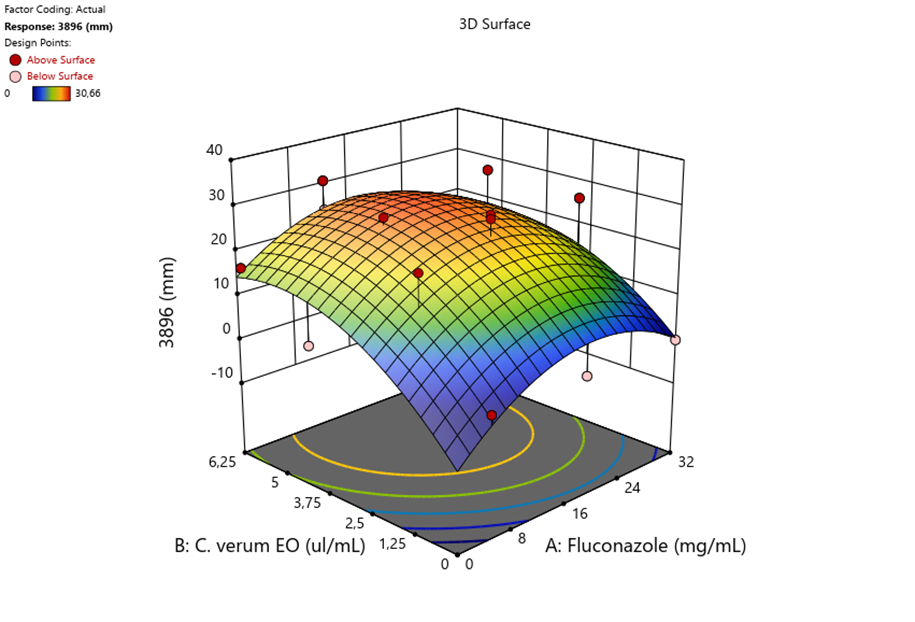 | 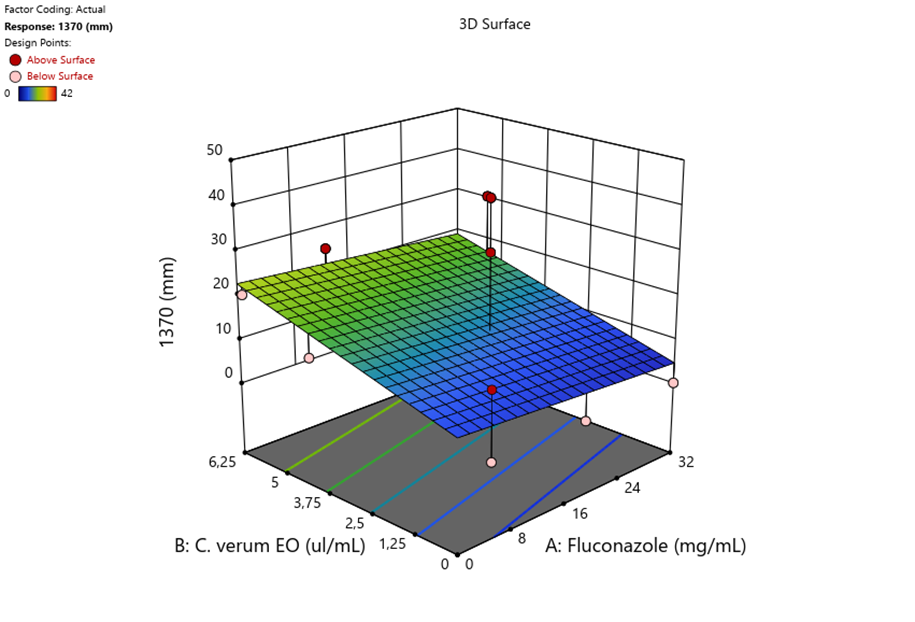 | 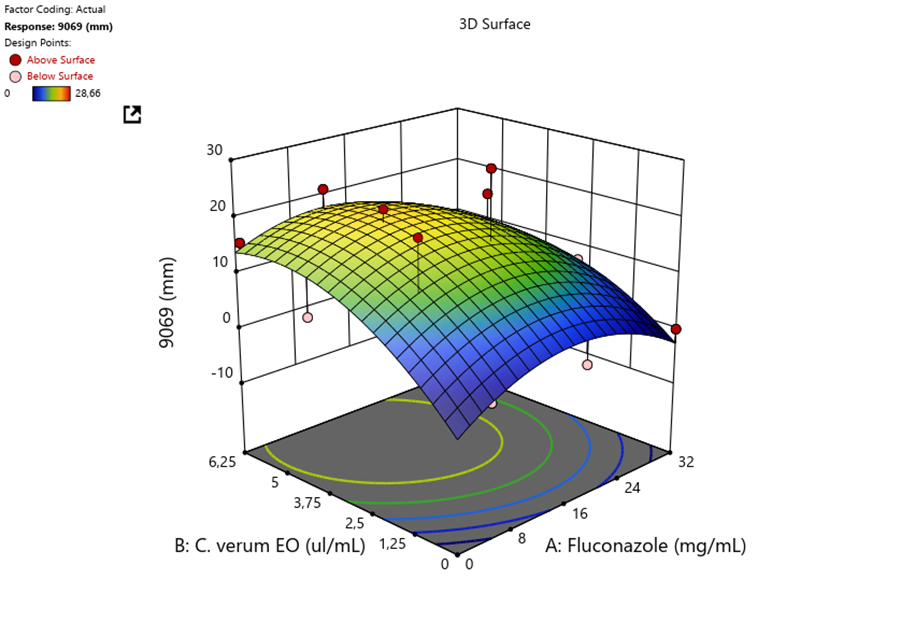 |
| 2851 | 3896 | 1370 | 9069 |
| 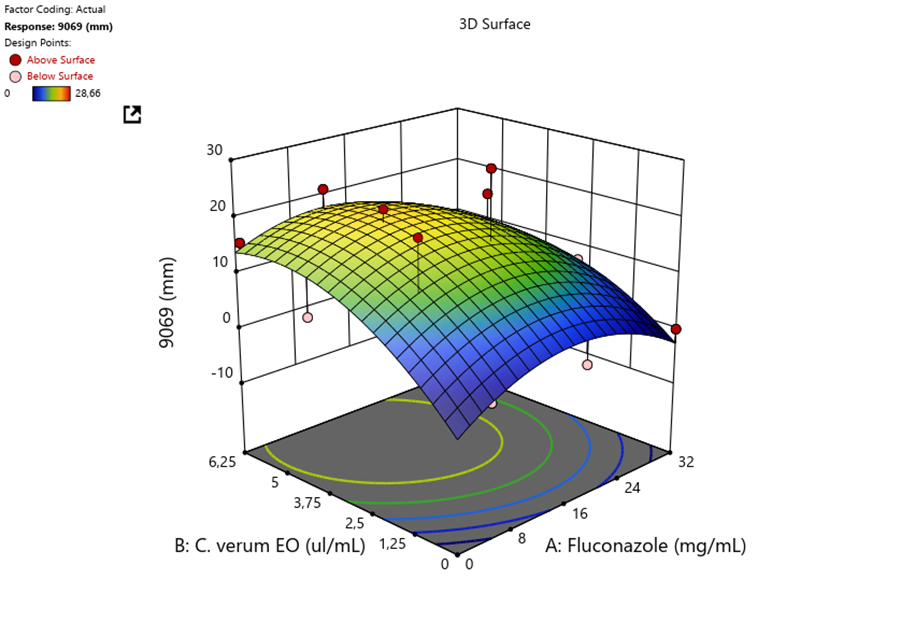 | 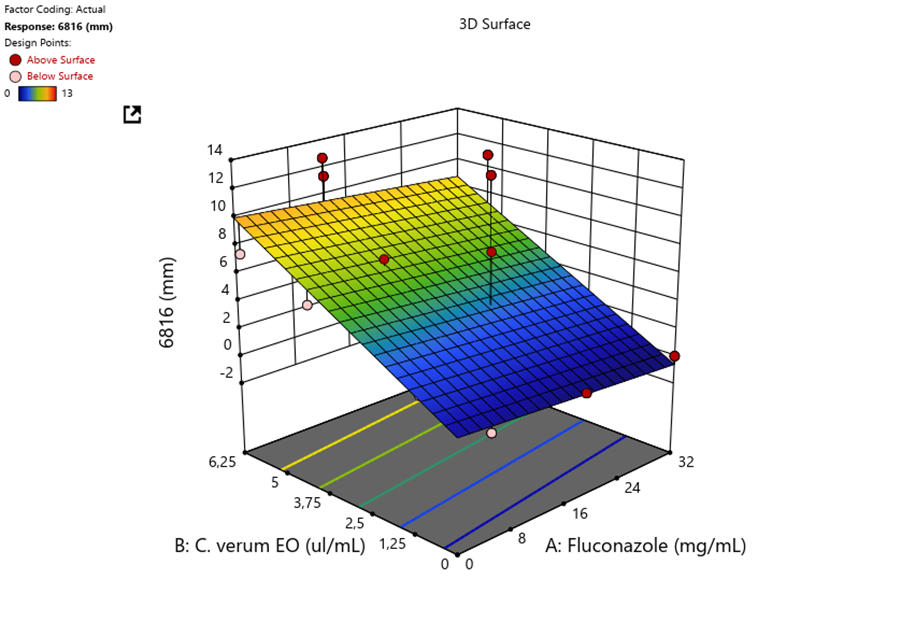 | 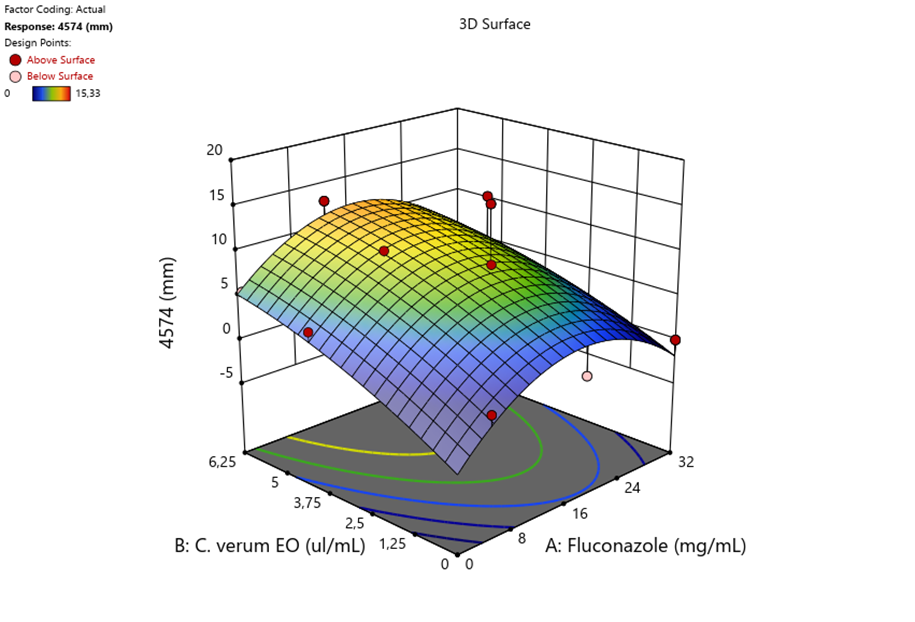 | 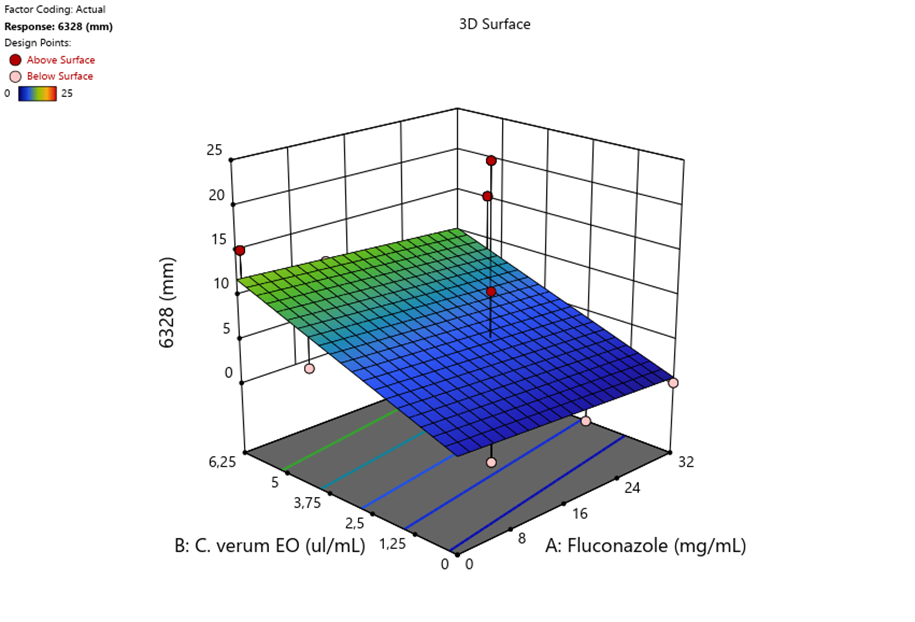 |
| 18519 | 6816 | 4574 | 6328 |

*Supplementary Figure S2.* Response surface analysis (RSM) of antifungal activity against *Candida* spp. based on *C. verum* EO and fluconazole concentrations

**Supplementary Table S16**. The percentages of the ability of *Candida* spp. strains to secrete hemolysins in the presence of EO, fluconazole, and their synergistic combination, or in the absence of treatment.

| **Hemolysin (%)** | *C. auris* DSM 21092 | | *C. albicans* ATCC 10231 | | *C.tropicalis* DSM 7524 | | *C. parapsilosis* ATCC 22019 | |
| --- | --- | --- | --- | --- | --- | --- | --- | --- |
|  | Mean | SD | Mean | SD | Mean | SD | Mean | SD |
| Cinnamon EO | 38,05263 | 7,443229 | 46,3333 | 11,78511 | 23,07692 | 18,84223 | 26,66667 | 9,42809 |
| Fluconazole | 52,63158 | 7,443229 | 48,3333 | 11,78511 | 30,76923 | 21,75713 | 10 | 8,164966 |
| Cinnamon EO+Fluconazole | 21,42105 | 7,443229 | 28,3333 | 21,18048 | 23,07692 | 18,84223 | 6,666667 | 12,47219 |
| Untreated | 100 | 7,443229 | 100 | 20,41241 | 100 | 39,22323 | 100 | 14,14214 |

**Supplementary Table S17.** The percentages of the ability of *Candida* spp. strains to secrete lipase in the presence of EO, fluconazole, and their synergistic combination, or in the absence of treatment.

| **Lipases (%)** | *C. auris* DSM 21092 | | *C. albicans* ATCC 10231 | | *C. tropicalis* DSM 7524 | | *C. parapsilosis* ATCC 22019 | |
| --- | --- | --- | --- | --- | --- | --- | --- | --- |
|  | Mean | SD | Mean | SD | Mean | SD | Mean | SD |
| Cinnamon EO | 25 | 8,838835 | 50 | 23,38536 | 73,33333 | 41,09609 | 33,33333 | 18,85618 |
| Fluconazole | 18,75 | 15,30931 | 43,75 | 35,35534 | 100 | 56,56854 | 33,33333 | 18,85618 |
| Cinnamon EO+Fluconazole | 31,25 | 8,838835 | 31,25 | 8,838835 | 26,66667 | 9,42809 | 33,33333 | 9,42809 |
| Untreated | 100 | 8,838835 | 100 | 8,838835 | 100 | 16,32993 | 100 | 16,32993 |

**Supplementary Table 18S.** The percentages of the ability of *Candida* spp. strains to secrete caseinase in the presence of EO, fluconazole, and their synergistic combination, or in the absence of treatment.

| **Caseinases (%)** | *C. auris* DSM 21092 | | *C. albicans* ATCC 10231 | | *C.tropicalis* DSM 7524 | | *C. parapsilosis* ATCC 22019 | |
| --- | --- | --- | --- | --- | --- | --- | --- | --- |
|  | Mean | SD | Mean | SD | Mean | SD | Mean | SD |
| Cinnamon EO | 36,66667 | 20,54805 | 54,54545 | 14,84539 | 62,06897 | 33,78607 | 53,125 | 19,26379 |
| Fluconazole | 26,66667 | 12,47219 | 60,60606 | 18,68004 | 62,06897 | 22,34738 | 43,75 | 17,67767 |
| Cinnamon EO+Fluconazole | 26,66667 | 24,94438 | 54,54545 | 37,11348 | 55,17241 | 34,13619 | 50 | 34,51675 |
| Untreated | 100 | 8,164966 | 100 | 12,85649 | 100 | 4,876598 | 100 | 4,419417 |

**Supplementary Table 19S**. The number of CFU/mL recovered from *G. mellonella* homogenates following the administration of EO, fluconazole and their combination.

|  |  |  |  |  |  |  |  |  |
| --- | --- | --- | --- | --- | --- | --- | --- | --- |
| **CFU/mL** | *C. auri*s DSM 21092 | | *C. albicans* ATCC 10231 | | *C. tropicalis* DSM 7524 | | *C. parapsilosis* ATCC 22019 | |
|  | Mean | SD | Mean | SD | Mean | SD | Mean | SD |
| Cinnamon EO | 2,666667 | 2,054805 | 2 | 0 | 2,666667 | 2,054805 | 0 | 0 |
| Fluconazole | 2 | 0,816497 | 1 | 0,816497 | 1,666667 | 0,471405 | 0 | 0 |
| Cinnamon EO+Fluconazole | 0 | 0 | 0,666667 | 0,942809 | 0 | 0 | 0 | 0 |
| Untreated | 23 | 5,715476 | 2 | 1,414214 | 1,333333 | 0,942809 | 5,666667 | 0,942809 |
